# The histamine receptor H1 acts as an alternative receptor for SARS-CoV-2

**DOI:** 10.1128/mbio.01088-24

**Published:** 2024-07-02

**Authors:** Fei Yu, Xiaoqing Liu, Hailan Ou, Xinyu Li, Ruxin Liu, Xi Lv, Shiqi Xiao, Meilin Hu, Taizhen Liang, Tao Chen, Xuepeng Wei, Zhenglai Zhang, Sen Liu, Han Liu, Yiqiang Zhu, Guangyan Liu, Tianyong Tu, Peiwen Li, Hui Zhang, Ting Pan, Xiancai Ma

**Affiliations:** 1 Medical Research Institute, Guangdong Provincial People’s Hospital (Guangdong Academy of Medical Sciences), Southern Medical University, Guangzhou, Guangdong, China; 2 Guangzhou National Laboratory, Guangzhou International Bio-Island, Guangzhou, Guangdong, China; 3 Institute of Human Virology, Zhongshan School of Medicine, Sun Yat-sen University, Guangzhou, Guangdong, China; 4 Shenzhen Key Laboratory of Systems Medicine for Inflammatory Diseases, Shenzhen Campus of Sun Yat-sen University, Shenzhen, Guangdong, China; 5 School of Medicine, South China University of Technology, Guangzhou, Guangdong, China; 6 Department of Breast Surgery, The Second Affiliated Hospital of Guangzhou Medical University, Guangzhou, Guangdong, China; 7 State Key Laboratory of Respiratory Disease, National Clinical Research Center for Respiratory Disease, Guangzhou Institute of Respiratory Health, the First Affiliated Hospital of Guangzhou Medical University, Guangzhou, Guangdong, China; 8 School of Biology and Biological Engineering, South China University of Technology, Guangzhou, Guangdong, China; 9 Department of Pathogen Biology, Shenyang Medical College, Shenyang, Liaoning, China; University of Calgary, Calgary, Canada

**Keywords:** SARS-CoV-2, receptor, HRH1, antihistamine, spike, viral entry

## Abstract

Numerous host factors, in addition to human angiotensin-converting enzyme 2 (hACE2), have been identified as coreceptors of severe acute respiratory syndrome coronavirus 2 (SARS-CoV-2), demonstrating broad viral tropism and diversified druggable potential. We and others have found that antihistamine drugs, particularly histamine receptor H1 (HRH1) antagonists, potently inhibit SARS-CoV-2 infection. In this study, we provided compelling evidence that HRH1 acts as an alternative receptor for SARS-CoV-2 by directly binding to the viral spike protein. HRH1 also synergistically enhanced hACE2-dependent viral entry by interacting with hACE2. Antihistamine drugs effectively prevent viral infection by competitively binding to HRH1, thereby disrupting the interaction between the spike protein and its receptor. Multiple inhibition assays revealed that antihistamine drugs broadly inhibited the infection of various SARS-CoV-2 mutants with an average IC50 of 2.4 µM. The prophylactic function of these drugs was further confirmed by authentic SARS-CoV-2 infection assays and humanized mouse challenge experiments, demonstrating the therapeutic potential of antihistamine drugs for combating coronavirus disease 19.

**IMPORTANCE:**

In addition to human angiotensin-converting enzyme 2, severe acute respiratory syndrome coronavirus 2 (SARS-CoV-2) can utilize alternative cofactors to facilitate viral entry. In this study, we discovered that histamine receptor H1 (HRH1) not only functions as an independent receptor for SARS-CoV-2 but also synergistically enhances ACE2-dependent viral entry by directly interacting with ACE2. Further studies have demonstrated that HRH1 facilitates the entry of SARS-CoV-2 by directly binding to the N-terminal domain of the spike protein. Conversely, antihistamine drugs, primarily HRH1 antagonists, can competitively bind to HRH1 and thereby prevent viral entry. These findings revealed that the administration of repurposable antihistamine drugs could be a therapeutic intervention to combat coronavirus disease 19.

## INTRODUCTION

The coronavirus disease 19 (COVID-19) pandemic, caused by severe acute respiratory syndrome coronavirus 2 (SARS-CoV-2), has persistently threatened public health (1, 2). In addition to SARS-CoV-2, influenza virus (IFV) and respiratory syncytial virus have concurrently circulated within human society. The development of potent polyvalent vaccines or antibody cocktails to prevent this “tripledemic” is urgently needed. However, both SARS-CoV-2 and IFV undergo multiple rounds of immune evasion and enhanced transmissibility, which significantly decreases the effectiveness of vaccines or antibodies targeting ancestral strains. Recently, a newly emerged SARS-CoV-2 Omicron lineage, designated JN.1, has started to prevail worldwide (3). Although the ACE2 binding affinity of JN.1 has slightly decreased, the increased immune evasion capability driven by intense immune pressure has raised a new round of public health concerns (4).

Human angiotensin-converting enzyme 2 (hACE2) is still the major receptor of SARS-CoV-2, although many mutations have been found in the receptor-binding domain (RBD) of spike (S) proteins across different SARS-CoV-2 mutants (5). However, mutations in regions other than the RBD have been found to affect the cellular tropisms of SARS-CoV-2 significantly. A representative example is the shift in the tropism of Omicron lineages. In addition to Omicron, the ancestral strain and other major variants of concern mainly infect and replicate within the lower respiratory tract, including the lungs, while Omicron lineages predominantly infect host cells within the upper respiratory tract, including the nose and throat (6, 7). Mutations within the S1/S2 cleavage boundary of the Omicron Spike not only attenuate furin-mediated cleavage but also cripple subsequent TMPRSS2-mediated spike activation, which potentially explains why Omicron lineages replicate inefficiently in TMPRSS2-expressing pulmonary epithelial cells (8–10). SARS-CoV-2 enters host cells through two major routes. After the recognition and binding of the spike protein to hACE2, the spike protein is primed and activated by cellular proteases such as TMPRSS2, followed by the fusion of viral and plasma membranes and subsequent release of viral genomic RNA (11). In cells lacking sufficient TMPRSS2, SARS-CoV-2 enters host cells via the endocytic pathway. Within endosomes, spike proteins are cleaved by cathepsin L (CTSL) to complete the activation, which triggers the fusion of viral and endosomal membranes (12–14).

Although hACE2 has been demonstrated to be the predominant receptor of SARS-CoV-2, the expression levels of hACE2 within the respiratory tract are relatively low compared to those in the kidney, heart muscle, and intestine (15). Multiple reports have revealed that SARS-CoV-2 can utilize accessory receptors to facilitate hACE2-dependent entry or use alternative receptors to perform hACE2-independent infection. High-density lipoprotein (HDL) scavenger receptor B type 1 promotes hACE2-dependent SARS-CoV-2 entry by indirectly interacting with cholesterol- and HDL-bound virions (16). Additionally, the neuropilin-1 (NRP1) receptor directly binds to the furin-cleaved S1 subunit of the spike protein and serves as the secondary cofactor for hACE2-dependent viral entry (17). Other coreceptors, including DC-SIGN, L-SIGN, SIGLEC1, vimentin, and ADAM9, have been found to facilitate viral attachment or interact with hACE2 to enhance hACE2-dependent infection (18–20). Another study showed that DC-SIGN and L-SIGN are capable of mediating SARS-CoV-2 entry by directly binding to the RBD (21). Multiple studies have revealed that CD147, AXL, KIM1, ASGR1, KREMEN1, LDLRAD3, CLEC4G, and TMEM106B function as alternative receptors of SARS-CoV-2 and enable hACE2-independent viral entry (22–27).

The identification of the hACE2 main receptor, as well as many “universal” coreceptors, has greatly diversified the use of bioactive compounds or repurposed drugs to prevent SARS-CoV-2 infection. However, directly targeting these receptors may also cause severe side effects (28). Therefore, identifying targets that can be utilized to design safe and effective drugs for SARS-CoV-2 prevention is still urgently needed (29). Interestingly, several antihistamine drugs, including clemastine, astemizole, azelastine, brompheniramine, and ebastine, which have been approved for treating allergy symptoms without side effects for decades, have been found to prevent SARS-CoV-2 infection or replication via protein-protein interaction analysis or drug library profiling (30–35). Another study revealed that the use of azelastine was associated with a reduced incidence of SARS-CoV-2 infection based on an analysis of over 219,000 electronic health records (36). Notably, all of the above antihistamines are histamine receptor H1 (HRH1) antagonists, suggesting that HRH1 could facilitate SARS-CoV-2 infection. HRH1 has been found to be widely expressed within respiratory tract tissues, including nasal and lung tissues (37, 38). Upon encountering allergens, released histamines can bind to HRH1, thereby triggering allergic rhinitis and allergic lung responses, while the competitive binding of antihistamine drugs to HRH1 alleviates histamine-induced allergies (39, 40).

In this study, we conducted an unbiased screening of a Food and Drug Administration (FDA)-approved drug library and found that all HRH1 antagonists could potently inhibit pseudotyped SARS-CoV-2 infection in susceptible cells by targeting the entry stage. These antihistamine drugs inhibited the entry of all the major viral mutants. Further mechanistic studies revealed that HRH1 acted as an hACE2-independent receptor for SARS-CoV-2 by directly binding to the N-terminal domain (NTD) of spike proteins. HRH1 also synergistically enhanced hACE2-dependent viral entry, mainly by binding to the hACE2 receptor. Authentic virus infection assays and transgenic hACE2 mouse challenge experiments further confirmed that antihistamine drugs could prevent SARS-CoV-2 infection. Our study provided compelling evidence that HRH1 acts as an alternative receptor for SARS-CoV-2. The administration of repurposable antihistamine drugs could be a potential treatment for COVID-19.

## RESULTS

### Antihistamine drugs inhibited pseudotyped SARS-CoV-2 infection

**Fig 1:**
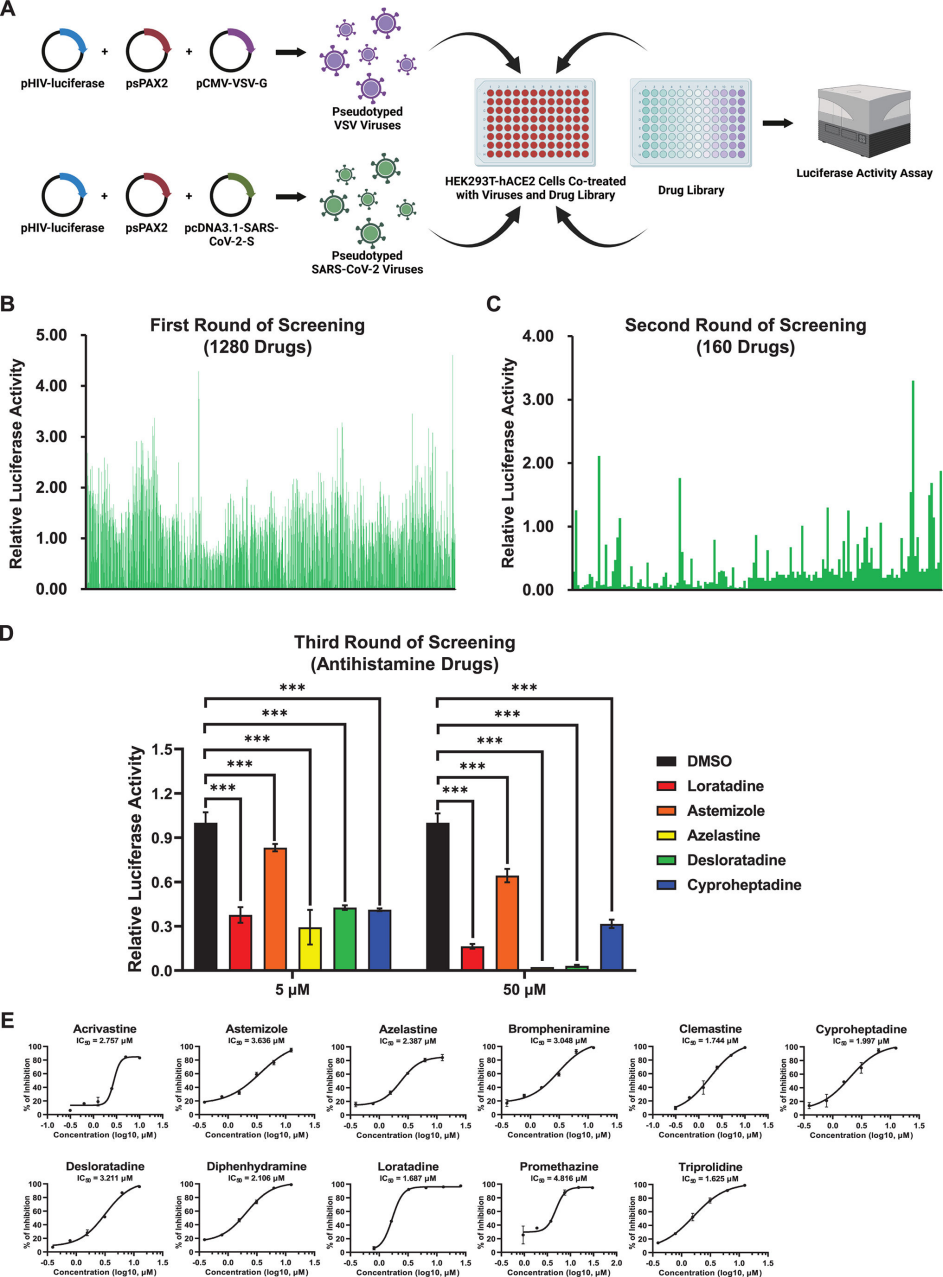
Antihistamine drugs inhibited pseudotyped SARS-CoV-2 infection. (**A**) Schematic of drug library screening for anti-SARS-CoV-2 drugs. pHIV-luciferase, psPAX2, and pcDNA3.1-SARS-CoV-2-S were cotransfected into HEK293T cells to produce pseudotyped SARS-CoV-2 viruses, while pseudotyped VSV viruses were generated by cotransfecting HEK293T cells with pHIV-luciferase, psPAX2, and pCMV-VSV-G. Different drugs (at 50 µM) from an FDA-approved drug library were premixed with pseudotyped viruses, followed by incubation with HEK293T-hACE2 cells. At 48 hpi, the cells were lysed, and luciferase activity was measured, which indicated the infectivity of the pseudotyped viruses upon co-treatment with each drug. (**B**) Relative luciferase activities of cells treated with pseudotyped SARS-CoV-2 viruses and 1,280 FDA-approved drugs. Measurements of the first round of screening were calculated by normalizing the luminescence units of each drug to those of DMSO. (**C**) Drugs that inhibited more than 75% of the number of pseudotyped SARS-CoV-2 viruses in the first round of screening (160 drugs) were selected, and the second round of screening was subsequently performed. The relative luciferase activity of each drug was calculated as described in panel **B**. (**D**) After two rounds of screening, five antihistamine drugs, namely, loratadine, astemizole, azelastine, desloratadine, and cyproheptadine, were selected for the third round of screening. Both 5 and 50 µM concentrations of each drug were tested. (**E**) Eleven commercially used antihistamine drugs at various concentrations were evaluated for their anti-infection effects. The inhibition of pseudotyped SARS-CoV-2 infection was determined by measuring relative luciferase activities. The half-maximal inhibitory concentration (IC50) of each drug was calculated and is shown in each panel. The data in panels **D** and **E** are presented as the means ± SEMs of biological triplicates. *P* values were calculated by one-way ANOVA with Dunnett’s multiple comparisons test. ***P < 0.001.

To systematically identify potential drugs that could inhibit SARS-CoV-2 infection, we screened a FDA-approved drug library that contained 1,280 widely used drugs (Fig. 1A). We utilized a pseudotyped virus infection system harboring an integrated *luciferase* gene driven by the EF-1α promoter (13). The expression level of luciferase indicated the infectivity of viruses upon infection of target cells. Each drug at a concentration of 50 µM was premixed with pseudotyped SARS-CoV-2 D614 viruses or VSV-G viruses, followed by incubation with HEK293T-hACE2 cells that stably overexpressed hACE2 receptors. At 48 h post-infection (hpi), the cells were lysed, and luciferase activity was measured (Fig. 1B). After the first round of screening, 160 drugs exhibiting greater than 75% inhibition of SARS-CoV-2 pseudotyped virus (PsV) infection were selected and subjected to the second round of screening (Fig. 1C). Remarkably, we found that nearly all antihistamine drugs present in the drug library inhibited SARS-CoV-2 PsV infection.

We conducted the third round of screening, specifically focusing on five antihistamine drugs, namely, loratadine, astemizole, azelastine, desloratadine, and cyproheptadine. The results showed that all of these antihistamines at both 5 and 50 µM potentially prevented SARS-CoV-2 PsV infection (Fig. 1D). Similar results have also been found in previous reports, which indicated that antihistamine drugs, including clemastine, astemizole, azelastine, brompheniramine, and ebastine, inhibited SARS-CoV-2 infection and replication (30–35). Antihistamine drugs are commonly used to treat histamine-induced allergies by competitively binding to histamine receptors without significant side effects (41). Two generations of antihistamine drugs, which vary in their ability to cross the blood-brain barrier, have been developed to alleviate symptoms of allergic rhinitis and allergic lung responses (42). To verify whether other antihistamine drugs could also inhibit SARS-CoV-2 infection, we evaluated six first-generation antihistamine drugs, namely, brompheniramine, clemastine, cyproheptadine, diphenhydramine, promethazine, and triprolidine, as well as five second-generation antihistamine drugs, namely, acrivastine, astemizole, azelastine, desloratadine, and loratadine. The PsV inhibition assay showed that all of these antihistamine drugs potentially inhibited SARS-CoV-2 PsVs infection, with half maximal inhibitory concentrations (IC50) ranging from 1.625 to 4.816 µM, while these drugs had minimal effects on VSV-G PsV infection even at a concentration of 200 µM (Fig. 1E; Fig. S1A). Collectively, our above results indicated that antihistamine drugs could be utilized to prevent SARS-CoV-2 infection.

### Antihistamine drugs prevented SARS-CoV-2 entry mainly by targeting HRH1

**Fig 2:**
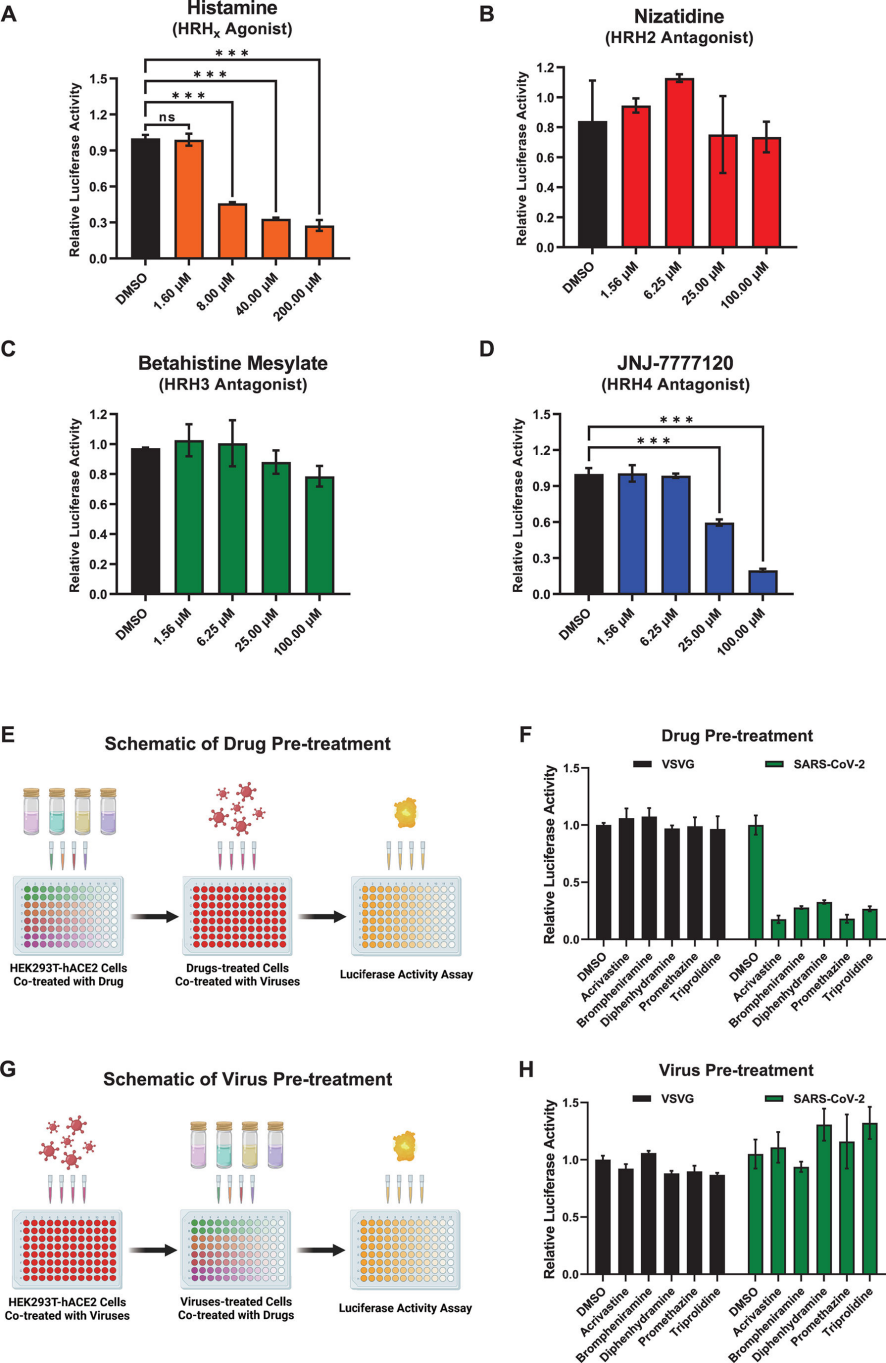
Antihistamine drugs prevented SARS-CoV-2 entry mainly by targeting HRH1. (**A**) HRH agonist histamines at various concentrations, including 1.60, 8.00, 40.00, and 200.00 µM, were premixed with pseudotyped SARS-CoV-2 viruses, followed by incubation with HEK293T-hACE2 cells. At 48 hpi, the cells were lysed, and the relative luciferase activity was measured. Measurements were calculated by normalizing the luminescence units of each group to those of the DMSO group. (**B**) The HRH2 antagonist nizatidine at different concentrations, including 1.56, 6.25, 25.00, and 100.00 µM, was premixed with pseudotyped SARS-CoV-2 viruses, followed by incubation with HEK293T-hACE2 cells. At 48 hpi, relative luciferase activities within each group were measured as described in panel **A**. (**C**) The effects of the HRH3 antagonist betahistine mesylate at concentrations of 1.56, 6.25, 25.00, and 100.00 µM on the entry of the pseudotyped SARS-CoV-2 virus were measured as described in panel **A**. (**D**) The HRH4 antagonist JNJ-7777120 was measured for its ability to inhibit pseudotyped SARS-CoV-2 entry, as described in panel **A**. (**E**) Schematic of the drug pretreatment assay. HEK293T-hACE2 cells were first treated with different antihistamine drugs. At 4 h post-treatment, the cells were further cotreated with SARS-CoV-2 or VSVG pseudotyped viruses. After another 48 h, the cells were lysed to measure the relative luciferase activity. (**F**) Five HRH1 antagonists at 10 µM were utilized to treat HEK293T-hACE2 cells as described in panel **E**, followed by infection of the cells with SARS-CoV-2 or VSVG PsVs. Luciferase activities within each group were measured and normalized to those in the DMSO group. (**G**) Schematic of the virus pretreatment assay. HEK293T-hACE2 cells were first infected with SARS-CoV-2 or VSVG PsVs, followed by coadministration of antihistamines at 4 hpi. Another 48 h later, the relative luciferase activity of the cells was measured. (**H**) HEK293T-hACE2 cells were infected with SARS-CoV-2 or VSVG PsVs as described in panel (**G**) and then cotreated with five HRH1 antagonists at 10 µM. Relative luciferase activities were measured and calculated by normalizing the luminescence units of each group to those of the DMSO group. The data in panels **A–D**, **F,** and **H** are presented as the means ± SEMs of biological triplicates. *P* values were calculated by one-way ANOVA with Dunnett’s multiple comparisons test. ***P < 0.001.

Based on therapeutic targets, antihistamine drugs can be classified into four subtypes: histamine receptor H1 antagonists, HRH2 antagonists, HRH3 antagonists, and HRH4 antagonists (41). Although all of their target receptors belong to the seven-transmembrane G-protein coupled receptor (GPCR) superfamily and respond to histamine stimulation, different types of histamine receptors are less conserved, sharing less than 25% protein sequence identity (Fig. S2A and B). Nevertheless, the protein sequences of human HRH1 (hHRH1) and mouse HRH1 (mHRH1) are highly conserved, with more than 75% identity. Interestingly, all of our above-tested antihistamine drugs, including those from the screened library, exclusively targeted HRH1, demonstrating the potential for HRH1-mediated SARS-CoV-2 infection. To verify whether the histamine agonist itself and other histamine receptor antagonists could universally prevent SARS-CoV-2 infection, we premixed SARS-CoV-2 D614 PsVs with histamines or three distinct types of antagonists targeting HRH2, HRH3, and HRH4. Subsequently, the compound/virus mixtures were incubated with HEK293T-hACE2 cells, and the relative luciferase activities were measured at 48 hpi. The results showed that histamines at concentrations of 8, 40, and 200 µM potently inhibited SARS-CoV-2 PsV infection, while neither the HRH2 antagonist nizatidine nor the HRH3 antagonist betahistine mesylate could prevent viral infection, even at a drug concentration of 100 µM (Fig. 2A through C). Interestingly, the HRH4 antagonist JNJ-7777120 inhibited viral infection at drug concentrations greater than 25 µM, suggesting that HRH4 might weakly mediate viral infection (Fig. 2D). These findings demonstrated that antihistamine drugs inhibited SARS-CoV-2 infection primarily by targeting HRH1 rather than HRH2, HRH3, or HRH4.

To determine the specific stage of viral infection targeted by HRH1 antagonists, we conducted drug or virus pretreatment assays. HEK293T-hACE2 cells were first treated with five antihistamine drugs, namely, acrivastine, brompheniramine, diphenhydramine, promethazine, and triprolidine, followed by infection with SARS-CoV-2 or VSV-G PsVs (Fig. 2E). The relative luciferase activities of the cells treated with each drug/virus combination were measured at 48 hpi. We found that all the drugs significantly prevented the infection of the SARS-CoV-2 PsVs regardless of the presence of the VSV-G PsVs upon drug pretreatment (Fig. 2F). In another group, HEK293T-hACE2 cells were first infected with SARS-CoV-2 or VSV-G PsVs. Subsequently, the cells were treated with different antihistamines at 4 hpi, after which the relative luciferase activity was measured at 48 h after drug treatment (Fig. 2G). The results showed that infection with SARS-CoV-2 or VSV-G PsVs was not affected by any drug upon virus pretreatment, which indicated that antihistamine drugs could not prevent SARS-CoV-2 infection upon the viral entry has been accomplished (Fig. 2H). Our results indicated that antihistamine drugs, mainly HRH1 antagonists, inhibited SARS-CoV-2 infection by targeting HRH1 at the viral entry stage.

### Antihistamine drugs inhibited SARS-CoV-2 entry in susceptible cell lines

**Fig 3:**
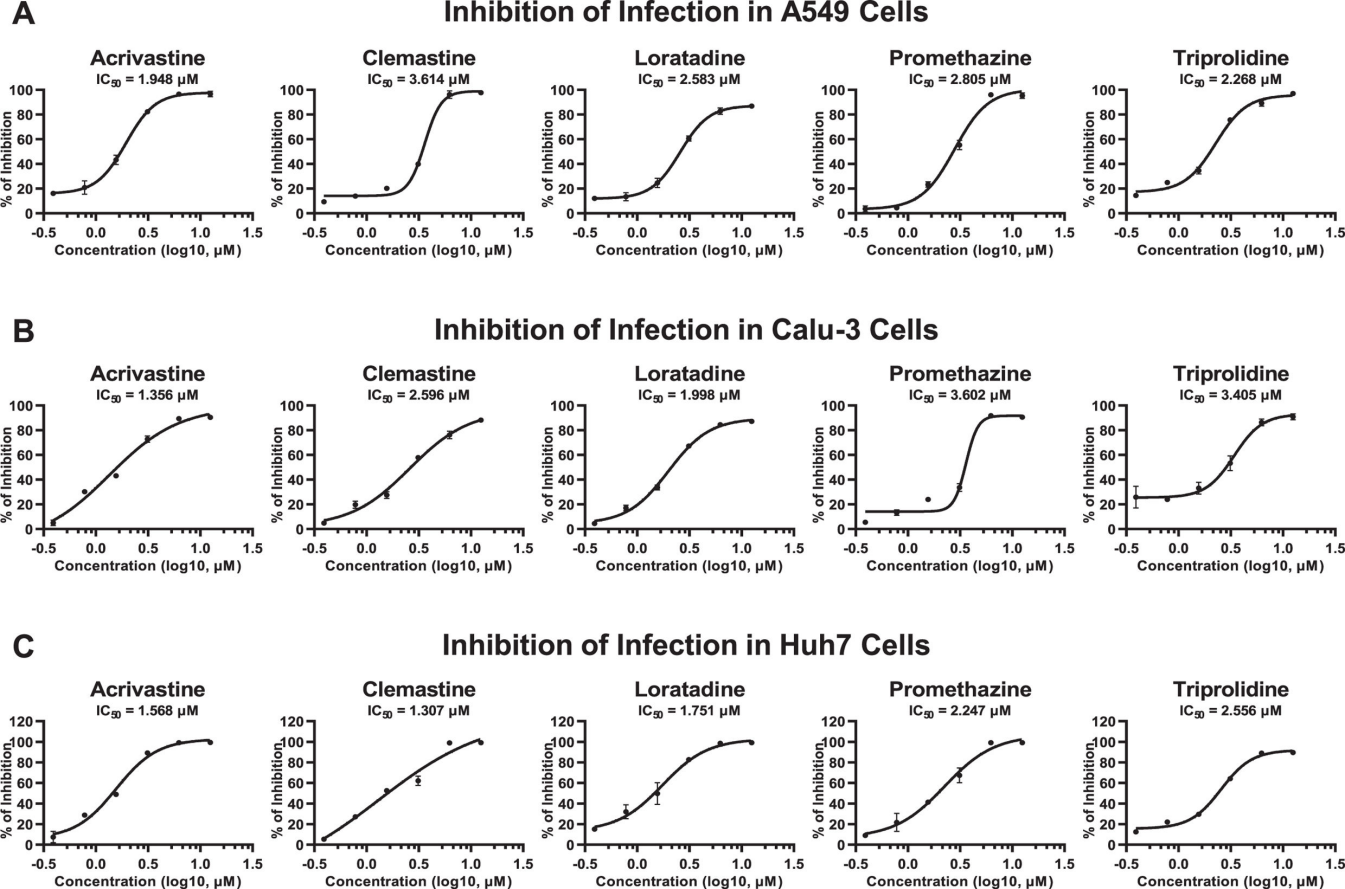
Antihistamine drugs inhibited SARS-CoV-2 entry in susceptible cell lines. (**A**) Five antihistamine drugs, namely, acrivastine, clemastine, loratadine, promethazine, and triprolidine, at serially diluted concentrations were premixed with SARS-CoV-2 D614 PsVs, followed by incubation with A549 cells. The IC50 for each drug was calculated based on the relative luciferase activity at 48 hpi. (**B**) The effects of five antihistamine drugs on the inhibition of SARS-CoV-2 D614 PsV entry into Calu-3 cells were evaluated as described in panel **A**. The IC50 of each drug in Calu-3 cells was determined. (**C**) The inhibition of SARS-CoV-2 D614 PsV entry by five antihistamine drugs in Huh7 cells was evaluated as described in panel **A**. The IC50 value for each drug in Huh7 cells was also calculated. The data in panels **A–C** are presented as the means ± SEMs of biological triplicates.

The primary sites of SARS-CoV-2 infection are the respiratory tract, including the nasal cavity, trachea, and lung, although the ACE2 expression levels within these tissues are relatively low (5, 15). Therefore, we utilized susceptible cell lines derived from these specific tissues further to evaluate the antihistamine drug-mediated inhibition of SARS-CoV-2 entry. SARS-CoV-2 PsVs were premixed with different concentrations of antihistamine drugs, followed by infection of human alveolar basal epithelial cell-derived A549 cells. We found that all five evaluated antihistamine drugs, namely, acrivastine, clemastine, loratadine, promethazine, and triprolidine, potently inhibited SARS-CoV-2 PsV entry into A549 cells, with IC50 values ranging from 1.948 to 3.614 µM (Fig. 3A). We also prepared drug/virus mixtures to treat human airway epithelial cell-derived Calu-3 cells. Similarly, acrivastine, clemastine, loratadine, promethazine, and triprolidine inhibited viral infection in Calu-3 cells, with IC50 values of 1.356, 2.596, 1.998, 3.602, and 3.405 µM, respectively (Fig. 3B). Additionally, we assessed drug-mediated viral inhibition in human hepatocyte-derived Huh7 cells, which are also commonly used as SARS-CoV-2-susceptible cells (11, 34, 43, 44). The results showed that antihistamine drugs also significantly inhibited SARS-CoV-2 PsV infection in Huh7 cells, with IC50 values less than 2.6 µM (Fig. 3C). Overall, our analysis indicated that HRH1 antagonists, which are subtypes of antihistamine drugs, were able to effectively inhibit SARS-CoV-2 entry into major susceptible cells, with an average IC50 value of 2.374 µM.

### HRH1 promoted SARS-CoV-2 entry in an ACE2-independent manner

**Fig 4:**
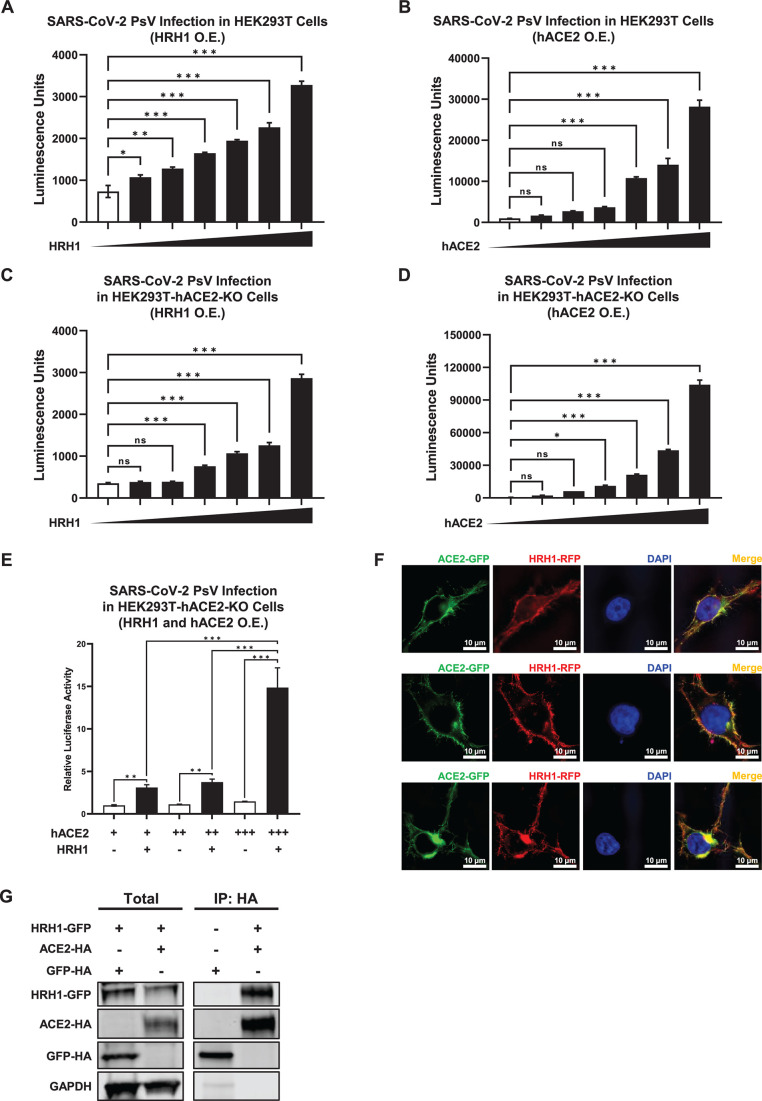
HRH1 promoted SARS-CoV-2 entry in an ACE2-independent manner. (**A**) HEK293T cells in 96-well plates were transfected with twofold serially diluted amounts of HRH1-expressing plasmids, ranging from 6.25 to 200 ng. At 24 h post-transfection, the cells were infected with SARS-CoV-2 D614 PsVs. After 48 hpi, the cells were lysed, and luminescence was measured. (**B**) HEK293T cells in 96-well plates were transfected with twofold serially diluted hACE2-expressing plasmids, ranging from 1.56 to 50 ng, followed by infection with SARS-CoV-2 D614 PsVs at 24 hpt. Luminescence units of cell lysates in each group were measured at 48 hpi. (**C**) HEK293T-hACE2-KO cells were transfected with different amounts of HRH1-expressing plasmids as in panel **A**, followed by infection with SARS-CoV-2 PsVs and measurement of luminescence units. (**D**) HEK293T-hACE2-KO cells were transfected with different amounts of hACE2-expressing plasmids as described in panel **B**, followed by infection with SARS-CoV-2 PsVs and measurement of luminescence. (**E**) HEK293T-hACE2-KO cells in 96-well plates were transfected with 0.39, 0.78, or 1.56 ng of hACE2-expressing plasmid. Another group of cells was cotransfected with 200 ng of HRH1-expressing plasmid. These cells were infected with SARS-CoV-2 PsVs at 24 hpt, and relative luciferase activities were measured at 48 hpi. (**F**) Green fluorescent protein (GFP)-tagged ACE2 and red fluorescent protein (RFP)-tagged HRH1 were co-overexpressed in HEK293T cells, followed by immunofluorescence assays utilizing structured illumination microscopy at 24 hpt. 4′,6-diamidino-2-phenylindole dihydrochloride (DAPI) was used to visualize DNA. IF images were captured at least three times. (**G**) HA-tagged ACE2 and HA-tagged GFP were co-overexpressed with GFP-tagged HRH1 in HeLa cells. Cells were lysed and immunoprecipitated (IP) with anti-HA beads, followed by western blotting for HA and Flag for both total (1/6 lysates) and IP samples. GAPDH was used as an internal control. The data in panels **A–E** are presented as the means ± SEMs of biological triplicates. *P* values in panels **A–D** were calculated by one-way ANOVA with Dunnett’s multiple comparisons test, while *P* values in panel **E** were calculated by one-way ANOVA with Tukey’s multiple comparisons test. *P < 0.05, **P < 0.01, and ***P < 0.001. The scale bars in panel **F** represent 10 µm.

As all the aforementioned antihistamine drugs that effectively inhibit SARS-CoV-2 infection are HRH1 antagonists, we speculated that HRH1 might facilitate SARS-CoV-2 entry. Therefore, we transiently overexpressed serially increased amounts of HRH1-expressing plasmids in HEK293T cells, followed by infection with SARS-CoV-2 D614 PsVs at 24 h post-transfection (hpt) (Fig. S3A). Our results showed that the overexpression of HRH1 significantly augmented viral infection, as indicated by increased numbers of luminescent units along with increased HRH1 expression (Fig. 4A). This phenomenon was similar to the hACE2 overexpression-mediated increase in the infectivity of SARS-CoV-2 PsVs in HEK293T cells (Fig. 4B; Fig. S3B). We also evaluated the HRH1-mediated enhancement of infection by additional viral mutants. The results showed that the overexpression of HRH1 in HEK293T cells also significantly enhanced the infection of several pseudotyped SARS-CoV-2 mutants, including D614G, Delta, BA.1, BA.2, BA.2.12.1, and BA.4, demonstrating the universal utilization of HRH1 for SARS-CoV-2 infection (Fig. S3C). These results indicated that HRH1 might act as a cofactor for SARS-CoV-2 entry. To exclude the possibility that endogenous hACE2 proteins within HEK293T cells might facilitate HRH1-mediated viral infection, we overexpressed HRH1 in hACE2-knockout HEK293T-hACE2-KO cells (Fig. S3D and E). We found that HRH1 also significantly enhanced SARS-CoV-2 PsV infection in the absence of hACE2 receptors (Fig. 4C). The re-introduction of hACE2 in HEK293T-hACE2-KO cells also rescued the infection of SARS-CoV-2 PsV (Fig. 4D). Interestingly, upon co-overexpression of both HRH1 and hACE2 in HEK293T-hACE2-KO cells, HRH1 significantly enhanced hACE2-mediated SARS-CoV-2 D614 PsV infection (Fig. 4E). This enhancement was also observed for other major SARS-CoV-2 variants, suggesting that HRH1 and hACE2 could synergistically facilitate SARS-CoV-2 entry (Fig. S3F). Immunofluorescence (IF) revealed that HRH1 colocalized with hACE2 on the cell membrane (Fig. 4F). Coimmunoprecipitation (CoIP) assays further showed that HRH1 could bind to hACE2 (Fig. 4G). Our results indicated that HRH1 promoted SARS-CoV-2 entry in an hACE2-independent manner. In addition, HRH1 enhanced hACE2-mediated viral entry.

### HRH1 bound to the NTD of the SARS-CoV-2 spike protein

**Fig 5:**
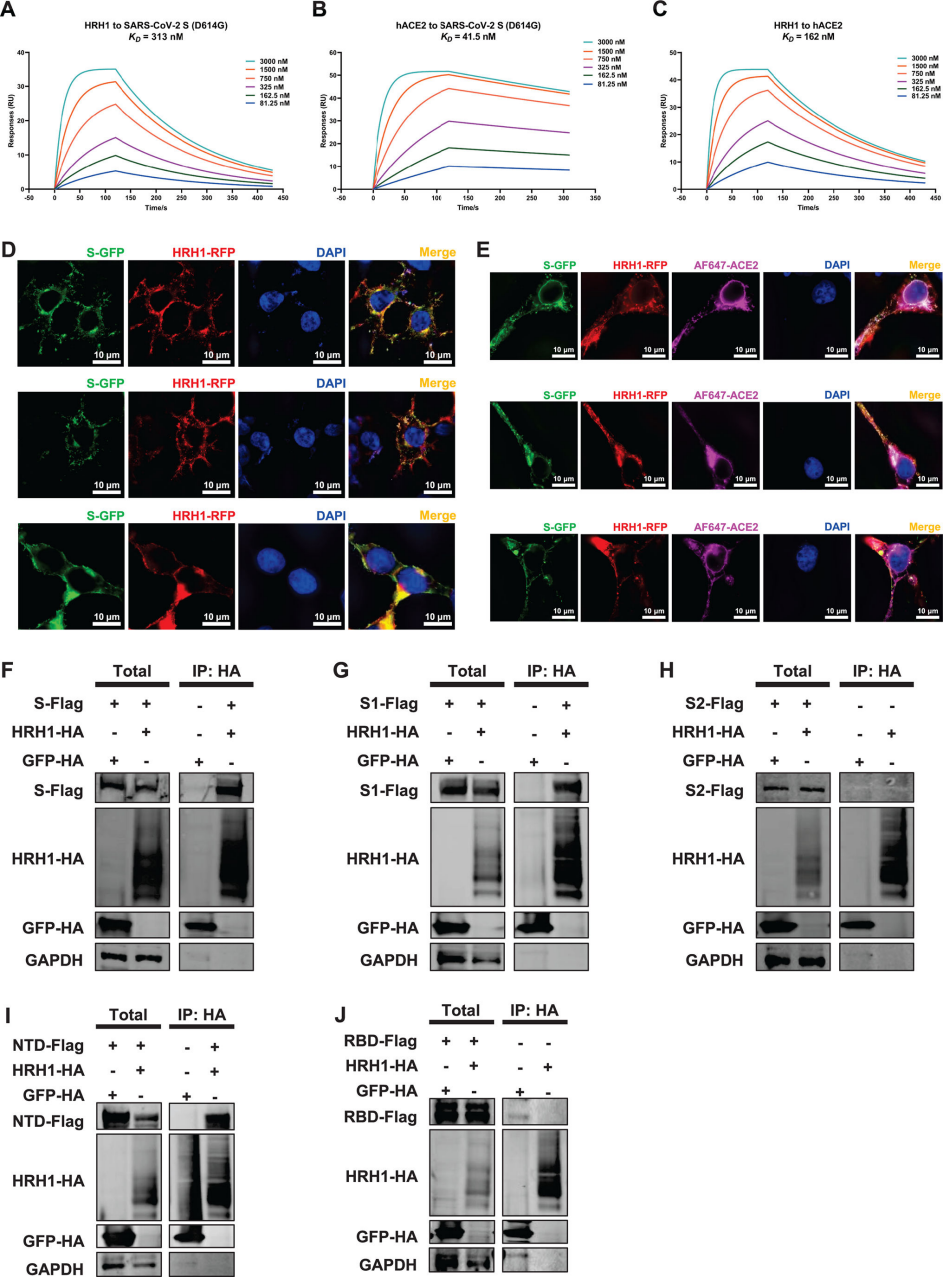
HRH1 bound to the NTD of the SARS-CoV-2 spike protein. (**A**) The binding affinity of HRH1 for SARS-CoV-2 S (D614G) was evaluated by SPR assay. Purified S proteins were immobilized on a CM5 sensor chip. Recombinant HRH1 proteins at concentrations of 81.25, 162.5, 325, 750, 1,500, and 3,000 nM were loaded on the chip and flowed over S proteins. The association rate (Ka) and dissociation rate (Kd) were measured. The equilibrium dissociation constant (KD) was calculated by dividing Ka by Kd (Kd/Ka). (**B**) The binding affinity of hACE2 for SARS-CoV-2 S (D614G) was evaluated as described in panel **A**, except that purified hACE2 proteins at concentrations of 81.25, 162.5, 325, 750, 1,500, and 3,000 nM were used as analytes. (**C**) The binding affinity of HRH1 for hACE2 was evaluated as described in panel **A**, except that purified hACE2 proteins were immobilized on the CM5 sensor chip. (**D**) Green fluorescent protein (GFP)-tagged S and red fluorescent protein (RFP)-tagged HRH1 were co-overexpressed in HEK293T cells. These cells were subjected to structured illumination microscopy (SIM) imaging to determine their distribution and colocalization at 24 hpt. (**E**) GFP-tagged S, RFP-tagged HRH1, and HA-tagged ACE2 were co-overexpressed in HEK293T cells, followed by SIM imaging with Alexa Fluor (AF) 647-tagged antibodies against ACE2 at 24 hpt. (**F**) HA-tagged HRH1 and HA-tagged GFP were co-overexpressed with Flag-tagged S in HeLa cells. At 48 hpt, the cells were subjected to IP with anti-HA beads and immunoblotted with antibodies against HA, Flag, and GAPDH. Both the 1/6 total lysates and IP samples were immunoblotted. (**G–J**) HA-tagged HRH1 and HA-tagged GFP were co-overexpressed with Flag-tagged S1, S2, NTD, or RBD in HeLa cells. IP and western blot assays were performed as described in panel **F**. The scale bars in panels **D** and **E** represent 10 µm. At least three samples were obtained for SIM imaging.

SARS-CoV-2 enters target cells through binding of viral spike proteins to cellular receptors (45). Both the receptor-binding domain and the N-terminal domain of S have been found to bind different receptors to enter susceptible cells (21, 23, 26, 46). To verify whether HRH1 could directly bind to S proteins, we utilized a surface plasmon resonance (SPR) assay to evaluate their interactions. The results showed that HRH1 was able to directly bind to SARS-CoV-2 D614G S with a binding constant of 313 nM, while hACE2 could strongly bind to S with a binding constant of 41.5 nM in our experimental assay (Fig. 5A and B; Fig. S4A). We also evaluated the interaction of HRH1 with hACE2. The SPR data indicated that HRH1 also directly bound to hACE2 with a binding constant of 162 nM, which was consistent with our previous finding that HRH1 coimmunoprecipitated and colocalized with hACE2 (Fig. 5C). Furthermore, our IF results showed that HRH1 colocalized with SARS-CoV-2 S and hACE2 on the cell membrane, similar to the colocalization of hACE2 and S (Fig. 5D and E; Fig. S4B).

HRH1 is a seven-transmembrane GPCR protein, while both hACE2 and SARS-CoV-2 S contain only one transmembrane domain (41, 45). Numerous attempts have been made to identify specific regions on HRH1 that bind to and colocalize with S proteins. However, neither truncations nor deletions of HRH1 could interact with S, suggesting that the binding ability of HRH1 to S relies on the convergence of multiple domains within HRH1. Therefore, we identified particular domains on the S protein that could bind to HRH1. First, we confirmed that HRH1 was able to coimmunoprecipitate with SARS-CoV-2 S, which was consistent with its *in vitro* binding ability quantified by SPR and that the colocalization of HRH1 was verified through IF (Fig. 5F). The S protein of SARS-CoV-2 is a single-transmembrane multidomain glycoprotein encompassing the S1 and S2 subunits, which are precleaved by the furin protease (47). Subsequently, we constructed S-derived mutants including Flag-tagged S1 and S2 domains (Fig. S4C). The results showed that S1, but not S2, was able to coimmunoprecipitate with HRH1 (Fig. 5G and H). S1 can be further partitioned into NTD and RBD subdomains, while the S2 region contains a fusion peptide, heptad repeat 1 (HR1), HR2, and transmembrane domain (48). To further elucidate which subdomain of S1 could bind to HRH1, we constructed Flag-tagged NTD- and RBD-expressing plasmids (Fig. S4C). Our results revealed that the NTD rather than the RBD bound to HRH1 (Fig. 5I and J). Collectively, our results confirmed that HRH1 is directly bound to SARS-CoV-2 S and hACE2. Notably, the NTD domain of S was the specific region that interacted with HRH1.

### Acrivastine and triprolidine inhibited SARS-CoV-2 mutant infection

**Fig 6:**
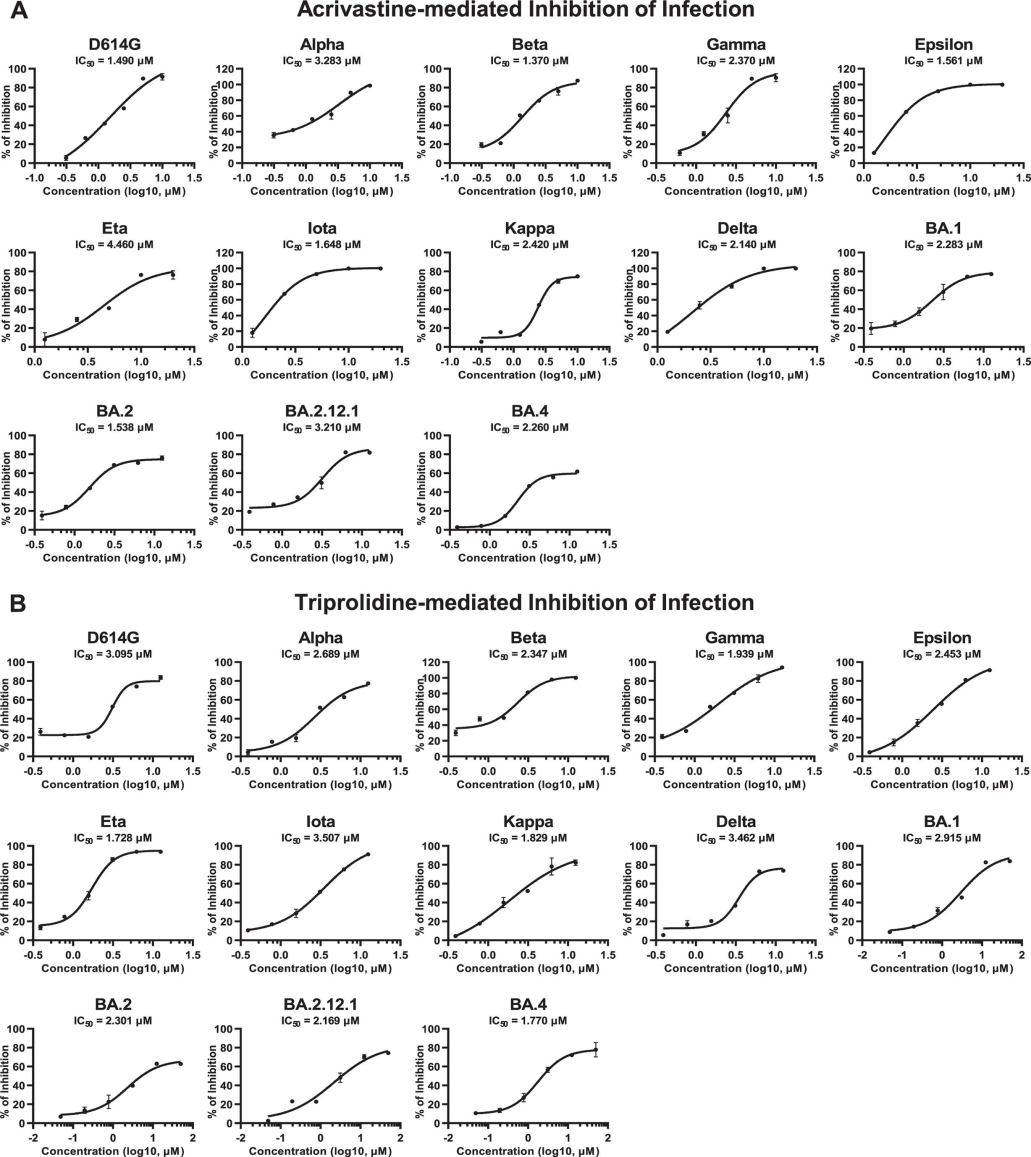
Acrivastine and triprolidine inhibited SARS-CoV-2 mutant infection. (**A**) Acrivastine at serially diluted concentrations was premixed with different SARS-CoV-2 PsVs, including D614G, Alpha, Beta, Gamma, Epsilon, Eta, Iota, Kappa, Delta, BA.1, BA.2, BA.2.12.1, and BA.4, followed by incubation with HEK293T-hACE2 cells. At 48 hpi, the IC50 value was calculated based on the relative luciferase activity of each viral mutant. (**B**) The inhibition of various SARS-CoV-2 PsV infections by triprolidine was evaluated as described in panel **A**. The IC50 value was calculated for each viral mutant. The data in panels **A** and **B** are presented as the means ± SEMs of biological triplicates.

Our above results showed that HRH1 could independently promote SARS-CoV-2 infection and enhance ACE2-dependent SARS-CoV-2 entry for various viral mutants. We speculated that antihistamine drugs targeting HRH1 might prevent infection by other SARS-CoV-2 mutants. Thus, we premixed acrivastine with multiple pseudotyped SARS-CoV-2 mutants, including D614G, Alpha, Beta, Gamma, Epsilon, Eta, Iota, Kappa, Delta, BA.1, BA.2, BA.2.12.1, and BA.4, followed by infection of HEK293T-hACE2 cells. The IC50 values of each drug/virus combination were determined based on relative luciferase activities, which were measured at 48 hpi. These inhibition assays showed that the infection of all the tested SARS-CoV-2 mutants could be prevented, with IC50 values ranging from 1.370 to 4.460 µM (Fig. 6A). Acrivastine is a second-generation antihistamine drug (49). We also evaluated whether the first-generation antihistamine drug triprolidine could inhibit the entry of pseudotyped SARS-CoV-2 mutants. Our results showed that triprolidine also inhibited the infection of various SARS-CoV-2 mutants, with IC50 values ranging from 1.728 to 3.507 µM (Fig. 6B). Similar results were also found for clemastine, loratadine, and promethazine, all of which were able to inhibit the infection of multiple pseudotyped SARS-CoV-2 mutants (Fig. S5A through C). Overall, our above results indicated that antihistamine drugs inhibited the infection of both ancestral SARS-CoV-2 and various emerged viral mutants with an average IC50 value of 2.370 µM, demonstrating their broad-spectrum inhibition of SARS-CoV-2 entry.

### Antihistamine drugs prevented SARS-CoV-2 infection in transgenic hACE2 mice

**Fig 7:**
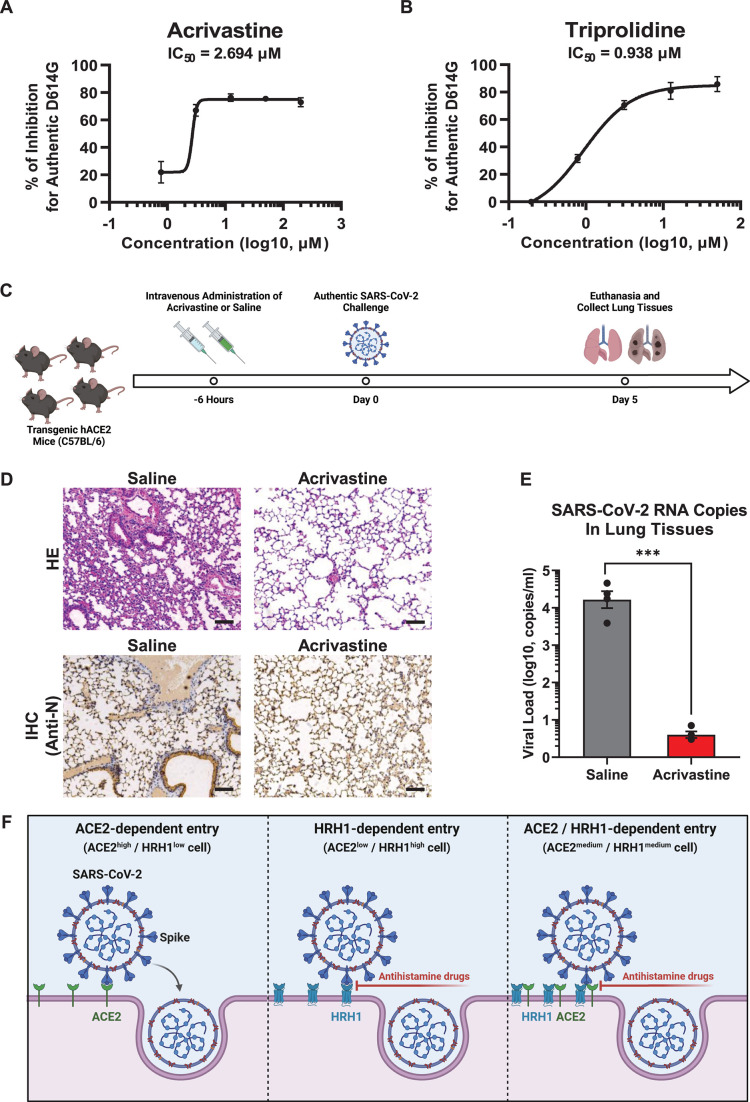
Antihistamine drugs prevented SARS-CoV-2 infection in transgenic hACE2 mice. (**A**) Acrivastine at serially diluted concentrations was premixed with authentic SARS-CoV-2 D614G viruses, followed by incubation with HEK293T-hACE2 cells. After 48 h, the viral RNA in the supernatants was extracted and quantified via RT-PCR-based detection. The IC50 value was calculated based on the relative number of viral RNA copies within each concentration. (**B**) The IC50 of triprolidine against the authentic SARS-CoV-2 D614G strain was measured and calculated as described in panel **A**. (**C**) Schematic of the animal infection assay. Four transgenic hACE2 mice (C57BL/6) were intravenously administrated with acrivastine at a dosage of 10 mg/kg of body weight. Mice in the control group were administrated with an equal volume of saline. Six hours later, all mice were intranasally challenged with 1 × 10^5^ focus-forming units of the SARS-CoV-2 D614G virus. On Day 5 post-infection, the mice were euthanized. Lung tissues were harvested and subjected to HE, immunohistochemistry (IHC), and viral quantification assays. (**D**) Lung tissues from both saline- and acrivastine-treated mice (challenged with authentic SARS-CoV-2) were analyzed by HE staining as well as IHC with antibodies against the SARS-CoV-2 N protein. (**E**) SARS-CoV-2 RNA copies in lung tissues were quantified via RT-PCR and are presented as log10 copies per milliliter (mL). (**F**) Schematic of ACE2- or HRH1-dependent SARS-CoV-2 entry. In ACE2^high^/HRH^low^ cells, SARS-CoV-2 viruses enter susceptible cells mainly by binding to ACE2 receptors through S proteins, while in cells with low ACE2 expression and high HRH1 expression (ACE2^low^/HRH1^high^ cells), SARS-CoV-2 viruses can utilize HRH1 as alternative receptors to enter target cells. In cells expressing both ACE2 and HRH1 (ACE2^medium^/HRH1^medium^ cells), SARS-CoV-2 can use both receptors to enter target cells. In addition, the presence of HRH1 could enhance ACE2-dependent viral entry. The data in panels **A** and **B** are presented as the means ± SEMs of biological triplicates. The data in panel **E** represent the mean ± SEM of biological quadruplicate samples. The *P* value in panel **E** was calculated by Student’s *t*-test. ***P < 0.001. The scale bars in panel **D** represent 100 µm.

As all of our previous screening and inhibition assays were conducted utilizing pseudotyped SARS-CoV-2, we wondered whether antihistamine drugs could inhibit authentic virus infection. We premixed authentic SARS-CoV-2 D614G viruses with different concentrations of acrivastine and triprolidine. These virus/drug mixtures were incubated with HEK293T-hACE2 cells for 48 h, after which the number of viral RNA copies within the supernatants was detected and quantified. The results showed that acrivastine inhibited authentic SARS-CoV-2 D614G infection with an IC50 value of 2.694 µM, while triprolidine prevented viral infection with an IC50 value of 0.938 µM (Fig. 7A and B). Ancestral SARS-CoV-2 viruses, including D614 and D614G variants, are unable to bind to mouse ACE2. Therefore, these viruses can hardly infect wild-type C57BL/6 or BALB/c mice. However, our pseudotyped virus infection assay showed that SARS-CoV-2 PsVs were able to utilize mHRH1 to infect HEK293T-hACE2-KO cells (Fig. S6A), which was consistent with our previous finding that hHRH1 and mHRH1 share high protein sequence identity (Fig. S2A and B). The IF results revealed that mHRH1 was able to colocalize with both SARS-CoV-2 S and hACE2, suggesting that mHRH1 could bind to the S protein to mediate viral entry and interact with hACE2 to enhance viral infection (Fig. S6B and C).

Having confirmed the ability of HRH1 antagonists to prevent authentic SARS-CoV-2 infection and the coalescence of mHRH1 with hACE2 to facilitate viral infection, we hypothesized that these antihistamine drugs might protect against authentic SARS-CoV-2 infection in mouse models. We utilized transgenic hACE2 mice (C57BL/6 background) that heterologously expressed human ACE2 in mouse lung tissues to conduct drug administration and viral challenge assays. We intravenously administrated acrivastine to each mouse at a dose of 10 mg/kg of body weight prior to viral infection (Fig. 7C). Mice in the control group were administrated with an equal volume of saline. Six hours later, each mouse was intranasally challenged with 1 × 10^5^ focus-forming units (FFUs) of the SARS-CoV-2 D614G virus. All mice were euthanized on Day 5 post-infection. Lung tissues from each mouse were harvested, and histopathology and immunohistochemistry analyses were performed. Hematoxylin and eosin (H and E) staining revealed severe lung lesions in lung tissues from SARS-CoV-2-infected mice in the control group, characterized by collapsed alveoli, thickened alveolar septa, and inflammatory cell infiltration (Fig. 7D). These pathological changes were not detected in lung tissues from mice pretreated with acrivastine. An immunohistochemistry assay with antibodies against SARS-CoV-2 nucleocapsid (N) proteins showed that lung tissues from saline-treated mice contained dispersed N-expressing epithelial cells, while acrivastine treatment effectively prevented this phenomenon (Fig. 7D). Furthermore, we also extracted and quantified SARS-CoV-2 RNA from lung tissues. The results revealed that lung tissues from saline-treated mice contained large amounts of viral RNA (2.28 × 10^4^ copies/mL on average), while those from acrivastine-treated mice harbored few copies of viral RNA (less than 10 copies/mL on average) (Fig. 7E). Taken together, our above results indicated that HRH1 antagonists, which are widely used antihistamine drugs, were able to inhibit authentic SARS-CoV-2 entry into susceptible cells and prevent SARS-CoV-2 infection in transgenic hACE2 mice.

## DISCUSSION

Human angiotensin-converting enzyme 2 and numerous cofactors have been found to promote SARS-CoV-2 infection (5, 16–27, 50). In this study, we provided compelling evidence that the histamine receptor H1 serves as an alternative receptor for SARS-CoV-2. The manipulation of HRH1 antagonists significantly inhibited SARS-CoV-2 infection. Based on all the results we reported above, we proposed a model of HRH1-mediated SARS-CoV-2 entry (Fig. 7F). In SARS-CoV-2-susceptible cells, which express high amounts of ACE2 and low levels of HRH1 (ACE2^high^/HRH1^low^ cells), viral spike proteins directly bind to cellular ACE2 receptors, thereby promoting the subsequent fusion of viral and cellular membranes and the release of viral genomic RNAs. However, in cells that express insufficient ACE2 but high levels of HRH1 (ACE2^low^/HRH1^high^ cells), SARS-CoV-2 can alternatively utilize HRH1 as a receptor to bind to spike proteins. Treatment with antihistamine drugs targeting HRH1 inhibited viral entry by preventing the binding of HRH1 to the spike protein. Remarkably, in susceptible cells that expressed medium levels of both ACE2 and HRH1 (ACE2^medium^/HRH1^medium^ cells), SARS-CoV-2 entered the cytoplasm in an ACE2/HRH1-dependent manner. HRH1 binds to ACE2 to bind to the viral spike protein and synergistically promotes ACE2-mediated viral entry. Manipulating antihistamine drugs that competitively bind to HRH1 could also abort the ACE2-mediated enhancement of viral infection.

In recent years, both computation-based virtual screening and cell-based experimental screening have been applied to discover repurposed drugs for combating COVID-19. Based on the SARS-CoV-2 life cycle, virus-targeted and host-directed antiviral drugs can be classified into four types: drugs targeting viral entry, replication, assembly, and exocytosis (51). Preventing virus attachment, endocytosis, or uncoating is the first line of defense against viral entry. However, the most available compounds that act directly on viruses during viral entry are spike-targeting monoclonal antibodies, which are expensive and narrow (52). To this end, multiple drugs directly targeting host factors upon SARS-CoV-2 infection have been identified through large-scale high-throughput screening (HTS), which has prompted the identification of novel entry mediators, including coreceptors and spike-activating proteases (30–34, 53, 54). We previously found that the glycopeptide antibiotic teicoplanin potently inhibited the entry of MERS-CoV, SARS-CoV, and SARS-CoV-2 through screening an FDA-approved drug library (55). Further mechanistic studies revealed that teicoplanin specifically inhibited the proteolytic activity of CTSL on viral spike proteins (13). Recently, we revealed that phenothiazine derivatives could inhibit the infection of distinct pseudotyped SARS-CoV-2 variants, as well as pseudotyped SARS-CoV and MERS-CoV, while another study further revealed that phenothiazines inhibited SARS-CoV-2 entry by blocking the binding of the spike protein to the cofactor NRP1 (56, 57). Notably, both previous screening studies and our screening findings revealed that multiple antihistamine drugs, mainly HRH1 antagonists, potently inhibited SARS-CoV-2 entry (30–35). We further demonstrated that HRH1 facilitated SARS-CoV-2 entry via direct binding to the NTD of the spike protein, which could be inhibited by the competitive binding of antihistamine drugs to HRH1. All of these discoveries indicate that large-scale, well-established drug screening identifies not only repurposable drugs to treat COVID-19 but also reveals alternative host factors to facilitate viral entry, which could further advance our understanding of the virus-host interaction landscape.

The repurposing of antihistamine drugs has also been applied in combating other infectious diseases (58). Through cell-based quantitative HTS of an approved drug library, He et al. (59) showed that the HRH1 antagonist chlorcyclizine HCl inhibited hepatitis C virus infection by targeting the late stage of viral entry. After screening an FDA-approved drug library, another group reported that two antihistamine drugs, carbinoxamine maleate and S-(+)-chlorpheniramine maleate, inhibited a broad spectrum of influenza A virus infections (60). Further mechanistic studies revealed that these drugs targeted the viral endocytosis stage rather than viral attachment. Schafer et al. (61) demonstrated that HRH1 antagonists, rather than antagonists targeting HRH2, HRH3, or HRH4, could inhibit the entry of filoviruses, including Ebola virus (EBOV) and Marburg virus. The authors speculated that these antihistamine drugs might bind directly to the EBOV glycoprotein based on docking studies. In addition to SARS-CoV-2, antihistamine drugs, including azelastine and clemastine, have been reported to inhibit the entry of other coronaviruses, such as pseudotyped SARS-CoV and MERS-CoV (32). Therefore, further studies should elucidate the mechanism of action of antihistamine drugs in preventing infections caused by a wide range of viruses and demonstrate the potential of universal coreceptor utilization of coronaviruses as well as many other pathogenic viruses.

Although we have provided compelling evidence that HRH1 can act as a receptor for SARS-CoV-2, several limitations exist in our study. Most of our inhibition assays against SARS-CoV-2 mutants were conducted utilizing pseudotyped virus infection experiments. Therefore, further studies should confirm the inhibitory effects of antihistamine drugs on various authentic SARS-CoV-2 variant-based cell infections and animal challenge assays. Although we provided evidence that HRH1 was able to bind directly to the viral spike protein, the interaction of which was inhibited by HRH1 antagonists, we cannot rule out the possibility that HRH1 antagonists may directly bind to the spike protein or ACE2. Drug-based SPR experiments could be conducted to elucidate their interactions in the future. In addition, our studies needed more data on the cocrystal structure of HRH1 and Spike due to the unsuccessful acquisition of full-length HRH1 proteins. Our animal model demonstrated that SARS-CoV-2 infection could be inhibited by antihistamine drugs, mainly by preventing the binding of the spike protein to mHRH1, but conducting similar drug administration and viral challenge experiments in conditional mHRH1-knockout mice would provide persuasive evidence to confirm the above hypothesis. As numerous coreceptors have been found to facilitate SARS-CoV-2 infection, performing viral infection and drug-blocking experiments in multi-receptor knockout cells would exclude the potential “off-target” effects of antihistamine drugs on other factors and validate the “on-target” effects of antihistamine drugs on HRH1-dependent viral entry.

Previous reports have indicated that SARS-CoV-2 infection can activate mast cells to release histamines, which further elevates proinflammatory cytokines, resulting in the formation of a COVID-19-related cytokine storm (62, 63). Therefore, antihistamine drugs could serve as immunomodulatory agents to alleviate the symptoms of COVID-19. Multiple clinical studies have also shown that the incorporation of antihistamine drugs to treat COVID-19 patients could reduce the duration of hospitalization and prevent the progression of severe symptoms (64–66). Consequently, antihistamine drugs are recommended for early treatment of COVID-19 (67, 68). Two clinical studies demonstrated that antihistamine drugs could quickly relieve long-term symptoms of COVID-19, including persistent rashes, multiorgan pain, intermittent anosmia, and chronic cardiovascular disorders, demonstrating the potential of antihistamine drugs for treating long-term COVID-19 (69, 70). Remarkably, our studies showed that HRH1 acted as an alternative receptor for SARS-CoV-2 by directly binding to the viral spike protein, while antihistamine drugs were able to competitively bind to HRH1, resulting in the prevention of SARS-CoV-2 entry. Based on our studies here and previous reports by others, antihistamine drugs could be utilized both as early prophylactic interventions to prevent SARS-CoV-2 infection and as late therapeutic countermeasures to mitigate long-term COVID-19 symptoms.

## MATERIALS AND METHODS

### Cell lines

The HEK293T (ATCC, CRL-3216), HeLa (ATCC, CCL-2), A549 (ATCC, CCL-185), Huh7 (JCRB, JCRB0403), and Vero E6 (ATCC, CRL-1586) cell lines were cultured in Dulbecco's modified Eagle medium (DMEM) (Thermo Fisher) supplemented with 10% fetal bovine serum (FBS) (Thermo Fisher), 100 units/mL penicillin, and 100 µg/mL streptomycin (Thermo Fisher). Calu-3 (ATCC, HTB-55) cells were maintained in minimum essential medium (MEM) (Thermo Fisher) supplemented with 10% FBS (Thermo Fisher), 100 units/mL penicillin, 100 µg/mL streptomycin (Thermo Fisher), and 55 µM β-mercaptoethanol (Thermo Fisher). All cells were cultured at 37°C and 5% CO_2_. These cells were tested for *Mycoplasma* contamination by a PCR-based assay (*Mycoplasma*-F: 5′-GGGAGCAAACAGGATTAGTATCCCT-3′; *Mycoplasma*-R: 5′-TGCACCATCTGTCACTCTGTTACCCTC-3′) and confirmed to be *Mycoplasma* free.

The HEK293T-hACE2 cell line was generated by overexpressing hACE2. Lentiviruses encoding hACE2 were used to infect wild-type HEK293T cells, followed by fluorescence-activated cell sorting for hACE2-positive cells. The successful construction of this cell line was confirmed by western blot and flow cytometry analysis of hACE2. The HEK293T-hACE2-KO cell line was generated by CRISPR-Cas9-mediated knockout. The sequence of sghACE2 (5′-TCAGTCCACCATTGAGGAAC-3′) was cloned and inserted into the lentiCRISPR v2 vector (Addgene plasmid # 52961). Lentiviruses targeting hACE2 were then packaged in HEK293T cells and reinfected with HEK293T cells to knock out endogenous hACE2. The monoclonal HEK293T-hACE2-KO cell line was obtained via a limiting dilution assay. Successful knockout of hACE2 was confirmed by both western blotting of hACE2 proteins and Sanger sequencing of *ACE2* genomic DNA.

### Viruses

Pseudotyped SARS-CoV-2 viruses and VSV-G viruses were packaged utilizing a lentivirus system. Briefly, sequences encoding the S domain of SARS-CoV-2 variants (including D614, D614G, Alpha, Beta, Gamma, Epsilon, Eta, Iota, Kappa, Delta, BA.1, BA.2, BA.2.12.1, and BA.4) and the glycoprotein (G) domain of VSV were cloned and inserted into the pcDNA3.1 vector. Lentiviruses expressing these proteins were packaged by cotransfecting the lentiviral construct pHIV-luciferase (Addgene plasmid # 21375), the packaging construct psPAX2 (Addgene plasmid # 12260), and the plasmid expressing the S or G protein into HEK293T cells. Supernatants containing these lentiviruses were harvested, infected with target cells, or stored at −80°C. The expression of luciferase indicated the infection of lentiviruses.

Authentic SARS-CoV-2 D614G viruses (GISAID: EPI_ISL_444969) were isolated from the sputum sample of a patient admitted to Guangzhou Eighth People’s Hospital. Vero E6 cells were utilized to propagate these viruses.

### Animal models

For authentic virus challenge experiments, 8-week-old specific-pathogen-free (SPF) transgenic hACE2 mice [C57BL/6JGpt-*H11^em1Cin(K18-hACE2)^*/Gpt] (strain no. T037657) were purchased from GemPharmatech Co., Ltd. These mice were housed in an SPF facility at the Laboratory Animal Center of Sun Yat-sen University.

### Drug library screening assay

A Food and Drug Administration-approved drug library (Topscience, L4200) containing 1,280 widely used drugs was used to conduct high-throughput screening for antiviral drugs. Pseudotyped SARS-CoV-2 D614 viruses or VSV-G viruses were packaged by transfecting HEK293T cells with the S- or G-expressing plasmid, the packaging plasmid psPAX2, and the lentiviral plasmid pHIV-luciferase. Approximately 1 × 10^6^ HEK293T-hACE2 cells were seeded in each well of 96-well plates. Each drug from the library at 50 µM was mixed with pseudotyped viruses and incubated with seeded cells at 24 h post-seeding. At 48 h post-infection, the cells were lysed, and a luciferase activity assay was performed. The expression level of lentivirus-driven luciferase indicated the infectivity of pseudotyped viruses upon cotreatment with each drug. The pseudotyped VSV-G virus infection assay was used as the negative control. After the first round of screening, drugs resulting in more than 75% inhibition of pseudotyped SARS-CoV-2 D614 viruses, which included 160 drugs, were selected for the second round of screening. After the second round of screening, five antihistamine drugs at 5 and 50 µM were specifically selected, and the third round of screening was subsequently performed. Each round of screening was repeated three times. Relative luciferase activity was calculated by normalizing the luminescence units of each drug to those of dimethyl sulfoxide (DMSO).

### Pseudotyped virus infection assay

Pseudotyped SARS-CoV-2 viruses, including D614, D614G, Alpha, Beta, Gamma, Epsilon, Eta, Iota, Kappa, Delta, BA.1, BA.2, BA.2.12.1, and BA.4, were packaged as described above. Antihistamine drugs at various dilutions were premixed with different pseudotyped viruses, followed by incubation with target cells, including HEK293T-hACE2, A549, Calu-3, and Huh7 cells. At approximately 48 hpi, the cells were lysed, and the relative luciferase activity was measured.

For the measurement of receptor- or candidate receptor-mediated viral infection, HEK293T cells or HEK293T-hACE2-KO cells were transfected with different amounts of HRH1-, hACE2-, or mHRH1-expressing plasmids at 24 h post-seeding. After 24 h, the cells were infected with SARS-CoV-2 D614 pseudotyped viruses. At 48 hpi, the cells were lysed, and the luciferase activity was measured. The infectivity of PsVs under various conditions is represented by luminescence units or relative luciferase activity. The expression of each protein was confirmed by western blotting.

### Drug pre- or post-treatment assay

To determine which stage the antihistamine drugs targeted, we conducted drug pretreatment or post-treatment assays. For the drug pretreatment assay, HEK293T-hACE2 cells were first treated with different antihistamines. After 4 h, the drug-treated cells were cotreated with VSV-G or SARS-CoV-2 D614 PsVs. After another 48 h, the cells were lysed to measure luciferase activity. For the drug post-treatment assay (also referred to as the virus pretreatment assay), HEK293T-hACE2 cells were first infected with VSV-G or SARS-CoV-2 D614 PsVs, followed by treatment with various antihistamine drugs at 4 hpi. Another 48 h later, the cells were lysed, and luciferase activity was measured.

### Coimmunoprecipitation

All CoIP assays were conducted with HeLa cells. We constructed different tag-conjugated constructs, including green fluorescent protein (GFP)-tagged HRH1, HA-tagged ACE2, Flag-tagged ACE2, HA-tagged GFP, and HA-tagged S. To identify the specific domain of S that binds to HRH1, we constructed Flag-tagged S, S1, S2, NTD, and RBD plasmids. HeLa cells were cotransfected with different tag-conjugated constructs. Approximately 48 h hpt, cells within 6 cm dishes were harvested and lysed with NP-40 lysis buffer [10 mM Tris-HCl (pH 7.5), 150 mM NaCl, 0.5% NP-40, 1% Triton X-100, 10% glycerol, 2 mM EDTA, 1 mM NaF, and 1 mM Na_3_VO_4_] supplemented with 1/100 protease inhibitor cocktail (Sigma-Aldrich). Cleared lysates were immunoprecipitated with anti-HA or anti-Flag beads based on the experimental setup. After 12 h of incubation with the beads at 4°C, the bead-enriched proteins were washed five times with ice-cold STN IP washing buffer [10 mM Tris-HCl (pH 7.5), 150 mM NaCl, 0.5% NP-40, and 0.5% Triton X-100]. Both IP samples and 1/6 total lysates were boiled with 5× protein SDS-PAGE loading buffer at 100°C for 10 min. Western blot assays with antibodies against GFP (Proteintech, 50430-2-AP), HA (MBL, PM020), Flag (MBL, M180-3), and GAPDH (Proteintech, 10494-1-AP) were conducted. Anti-GAPDH was used as the internal reference. The membranes were further incubated with secondary antibodies, including 680RD goat anti-mouse IgG (LI-COR Biosciences, 926-68070) and 800CW goat anti-rabbit IgG (LI-COR Biosciences, 926-32211), followed by development with an Odyssey M Imager (LI-COR Biosciences) and analysis with Image Studio Lite Ver 5.0 (LI-COR Biosciences).

### Immunofluorescence

To characterize the cellular localization and distribution of different proteins, including hACE2, S, HRH1, and mHRH1, we constructed different fluorescent protein-tagged constructs. Both green fluorescent protein and red fluorescent protein (RFP) were utilized. These constructs contained GFP-tagged ACE2, GFP-tagged S, GFP-tagged mHRH1, RFP-tagged HRH1, RFP-tagged ACE2, and RFP-tagged S. The immunofluorescence assay was conducted by cotransfecting HEK293T cells with both a GFP-tagged protein-expressing construct and an RFP-tagged protein-expressing construct. At approximately 24 hpt, the cells were fixed with 4% paraformaldehyde for 10 min, followed by permeabilization with 0.2% Triton X-100 for another 10 min. Then, the cells were incubated with 4′,6-diamidino-2-phenylindole dihydrochloride for 10 min to dye the DNA.

The prepared IF samples could be stored at 4°C for more than 2 weeks or were imaged directly via structured illumination microscopy (SIM). SIM images were captured on an Eclipse Ti inverted microscope equipped with a CFI Apo TIRF objective (1.49 NA, oil immersion). Additional equipment included NIS-Elements AR software, an sCMOS camera (Hamamatsu Flash 4.0, 6.5 μm × 6.5 µm pixel size), and four lasers (SIM 405, SIM 488, SIM 561, and SIM 647). Images were captured with 512 × 512 resolution and reconstructed to finalize the SIM image with 1,024 × 1,024 resolution. The resolutions of the reconstructed SIM images were 115 nm in lateral resolution and 300 nm in axial resolution. Fifteen images (five phases, three angles, 3D-SIM mode) were captured for each focal plane, reconstructed, and analyzed with the N-SIM module of the NIS-Elements AR software (Nikon).

### Protein expression and purification

To determine the interactions between HRH1, hACE2, and S, we expressed and purified the hACE2 and SARS-CoV-2 S (D614G) proteins *in vitro*. We also attempted to express HRH1 many times but failed. Thus, recombinant HRH1 proteins were purchased directly (AtaGenix, ATEP02127HU). Only the extracellular domains (ECDs) of hACE2 and S were expressed. The N-terminal signal peptides of both constructs were substituted with the following SP: MGILPSPGMPALLSLVSLLSVLLMGCVA, while the C-termini of both constructs were coexpressed with a 6× His tag. Additionally, eight substitution mutations, R683A, R685A, F817P, A892P, A899P, A942P, K986P, and V987P, were introduced into S-ECD to stabilize the prefusion status of S, the construct of which was named S-8M-ECD. Plasmids expressing hACE2-ECD and S-8M-ECD were transiently transfected into HEK293F cells. Approximately 7–10 days later, supernatants containing secretory proteins were harvested, and 6× His-tagged target proteins were purified with Ni-NTA agarose. Enriched proteins were washed with Tris buffer containing low concentrations of imidazole and eluted with Tris buffer containing high concentrations of imidazole. The eluted proteins were concentrated, and the buffer was replaced with conventional Tris buffer without imidazole. Protein purities were confirmed by both Coomassie blue staining and western blotting, while protein concentrations were determined by the bicinchoninic acid assay.

### Surface plasmon resonance

The binding affinities of HRH1 for hACE2 and SARS-CoV-2 S (D614G) were determined by surface plasmon resonance with a Biacore 8K^+^ instrument (Cytiva). Briefly, the aforementioned purified hACE2-ECD and S-8M-ECD proteins were immobilized on Flow Cell 2 (FC2) of the CM5 sensor chip utilizing an amine coupling kit. Flow Cell 1 (FC1), which was not loaded with ligand proteins, was treated as the reference surface. Serially diluted HRH1 proteins at concentrations ranging from 81.25 to 3,000 nM were injected over both FC1 and FC2 at a flow rate of 30 µL/min. For each cycle, the contact time was set to 120 s, while the dissociation time was set to 300 s. The binding affinity of hACE2 for S was also monitored as a positive control. The concentration range, flow rate, contact time, and dissociation time of hACE2-ECD were the same as those of the HRH1 proteins. Response units (RUs) were calculated by subtracting responses of the reference channel (FC1) from responses of the active channel (FC2). Adjusted RUs were fitted to a 1:1 binding model utilizing Biacore insight evaluation software version 4.0.8.19879 (Cytiva). Both the association rate (“on rate”, Ka) and dissociation rate (“off rate”, Kd) were measured and analyzed. The equilibrium dissociation constant (“binding constant”, KD) was calculated by dividing Ka by Kd (Kd/Ka).

### Authentic virus infection assay

The inhibitory effects of the antihistamines were confirmed via an authentic SARS-CoV-2 infection assay. HEK293T-hACE2 cells were cotreated with SARS-CoV-2 D614G viruses (GISAID: EPI_ISL_444969) and serially diluted antihistamine drugs, including acrivastine and triprolidine. At approximately 48 hpi, the supernatants from each group were collected, and total RNA was extracted utilizing an RNeasy Mini Kit (QIAGEN, 74104). The number of viral RNA copies within each group was quantified with a one-step SARS-CoV-2 RNA detection kit (PCR-Fluorescence Probing) (Da An Gene Co., DA0931). Primers and probes targeting the SARS-CoV-2 *nucleocapsid* (*N*) were utilized for quantification. N-F: 5′-CAGTAGGGGAACTTCTCCTGCT-3′. N-R: 5′-CTTTGCTGCTGCTTGACAGA-3′. N-P: 5′-FAM-CTGGCAATGGCGGTGATGCTGC-BHQ1-3'. RT-PCR experiments were repeated in biological triplicate. The half maximal inhibitory concentration (IC50) of acrivastine or triprolidine against SARS-CoV-2 D614G viruses was calculated via GraphPad Prism 9.0. All the above authentic virus-related experiments, including infection, RNA extraction, and quantification, were performed at the biosafety level 3 (BSL-3) facility of Sun Yat-sen University.

### Animal infection assay

Transgenic hACE2 mice, which were generated and purchased from GemPharmatech Co., Ltd. (strain no. T037657), were used for the animal infection and drug inhibition assays. Briefly, four 8-week-old SPF hACE2 mice were intravenously administrated with acrivastine (dissolved in saline) at a dosage of 10 mg/kg of body weight. Another four mice were intravenously administrated with an equal volume of saline. Approximately 6 h post-administration, all mice were intranasally challenged with 1 × 10^5^ FFUs of SARS-CoV-2 D614G virus (GISAID: EPI_ISL_444969). Five days later, the mice were euthanized. Lung tissues were collected for histopathology analysis, immunohistochemistry analysis, and viral RNA quantification. SARS-CoV-2 viral RNA copies in lung tissues were quantified utilizing a one-step SARS-CoV-2 RNA detection kit (PCR-Fluorescence Probing) (Da An Gene Co., DA0931). The primers and probes used to amplify SARS-CoV-2 *N* RNAs are described above. For each mouse, RT-PCR assays were performed in technical triplicate. For each group, RT-PCR experiments were conducted in biological quadruplicate. All the authentic virus-related experiments, including mouse challenge, euthanasia, and dissection, were performed at the BSL-3 facility of Sun Yat-sen University.

### Histopathology and immunohistochemistry

To evaluate the effects of viral challenge and drug treatment on mice, lung tissues from each mouse were collected, and both histopathology and immunohistochemistry analyses were performed (Nanjing FreeThinking Biotechnology Co., Ltd.). One lung lobe from each mouse was completely fixed with 4% paraformaldehyde, followed by paraffin embedding. Lung sections 3–4 µm in size were segmented, followed by staining with hematoxylin and eosin. Other groups of lung sections were deparaffinized and rehydrated with xylene and gradient alcohol. Citric acid buffer (pH 6.0) was used to retrieve antigens, followed by quenching with 3% H_2_O_2_. The samples were blocked with BSA and incubated with anti-SARS-CoV-2 N antibodies (Sino Biological, 40143-T62) for 24 h at 4°C. HRP-conjugated secondary antibodies (Sino Biological, SSA004) were used to label N-specific cells. The samples were further stained with 3,3′-diaminobenzidine and hematoxylin, followed by dehydration with gradient ethanol. Each sample was covered with neutral balsam. Images of each lung tissue sample were acquired utilizing an HS6 microscope (Sunny Optical Technology Co., Ltd.).

### Statistical analysis

All the statistical analyses in this study were conducted with GraphPad Prism 9.0 or Microsoft Excel. The statistical details, including the statistical tests used, exact values of the sample size, mean values, standard errors of the mean (SEMs), and *P* values, are provided in the main text, figures, methods, and figure legends. Biological data from triplicate and quadruplicate samples are presented as the mean ± SEM. Normally distributed data were analyzed by Student’s *t* test. Differences in the means of groups that were split by one independent variable were analyzed by one-way ANOVA with Tukey’s multiple comparisons test or Dunnett’s multiple comparisons test. Differences in the means of two independent variables between groups were analyzed by two-way ANOVA with Tukey’s multiple comparisons test or Dunnett’s multiple comparisons test. Values of *P* ≥ 0.05 were considered not statistically significant and are represented as “ns”. Values of *P* < 0.05 were considered to indicate statistical significance and are represented as single asterisks (*). Values of *P* < 0.01 were considered to be more statistically significant and are represented as double asterisks (**). Values of *P* < 0.001 were considered to be the most statistically significant and are represented as triple asterisks (***).

## Data Availability

The plasmids encoding the spike proteins of SARS-CoV-2 and the corresponding mutants, truncated spike mutants, hACE2, hHRH1, and mHRH1 are available from the corresponding authors upon request. Pseudotyped SARS-CoV-2 viruses, the HEK293T-hACE2 cell line, and the HEK293T-hACE2-KO cell line will be provided upon executing a material transfer agreement with inquiries directed to Prof. Xiancai Ma. Further information and requests for resources and reagents should be directed to and will be fulfilled by corresponding author Xiancai Ma (ma_xiancai@gzlab.ac.cn).

## SUPPLEMENTAL MATERIAL

Supplemental figures mbio.01088-24-s0001.docx Figures S1 to S6.

ASM does not own the copyrights to Supplemental Material that may be linked to, or accessed through, an article. The authors have granted ASM a non-exclusive, world-wide license to publish the Supplemental Material files. Please contact the corresponding author directly for reuse.
