# Supplementary material for: The histamine receptor H1 acts as an alternative receptor for SARS-CoV-2
Source: mBio. 2024 Jul 2;15(8):e01088-24. doi: 10.1128/mbio.01088-24 (PMC11324024; doi:10.1128/mbio.01088-24)
Supplement: Supplemental figures — Figures S1 to S6. [file mbio.01088-24-s0001.docx]

**Supplemental Figures**

**Fig. S1**


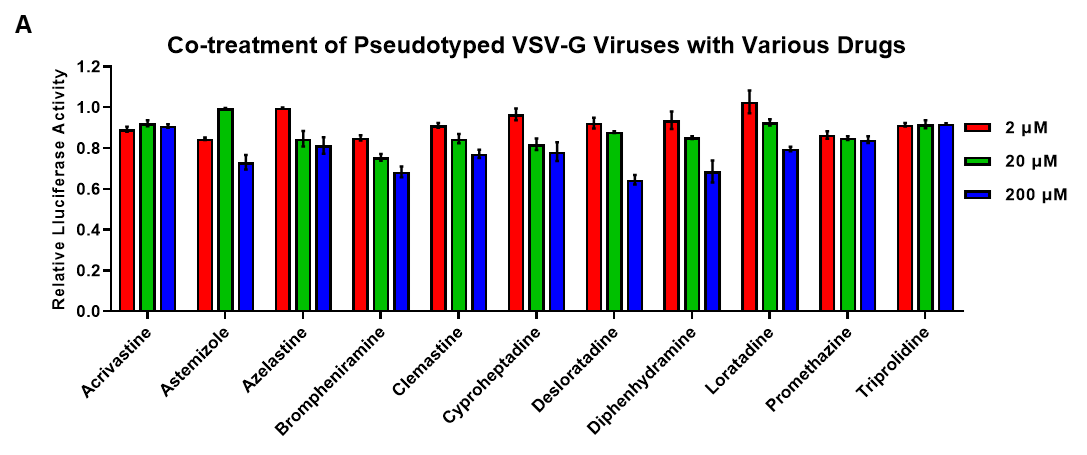


**Fig. S1. Antihistamine drugs did not inhibit the infection of pseudotyped VSV-G viruses.**

(A) Eleven commercially used antihistamine drugs (at 2, 20 and 200 μM), including acrivastine, astemizole, azelastine, brompheniramine, clemastine, cyproheptadine, desloratadine, diphenhydramine, loratadine, promethazine and triprolidine, were premixed with pseudotyped VSV-G viruses. Subsequently, these drug/virus mixtures were incubated with HEK293T-hACE2 cells. At 48 h posttreatment, the cells were lysed, and the relative luciferase activity was measured. The data are presented as the means ± SEMs of biological triplicates.

**Fig. S2**


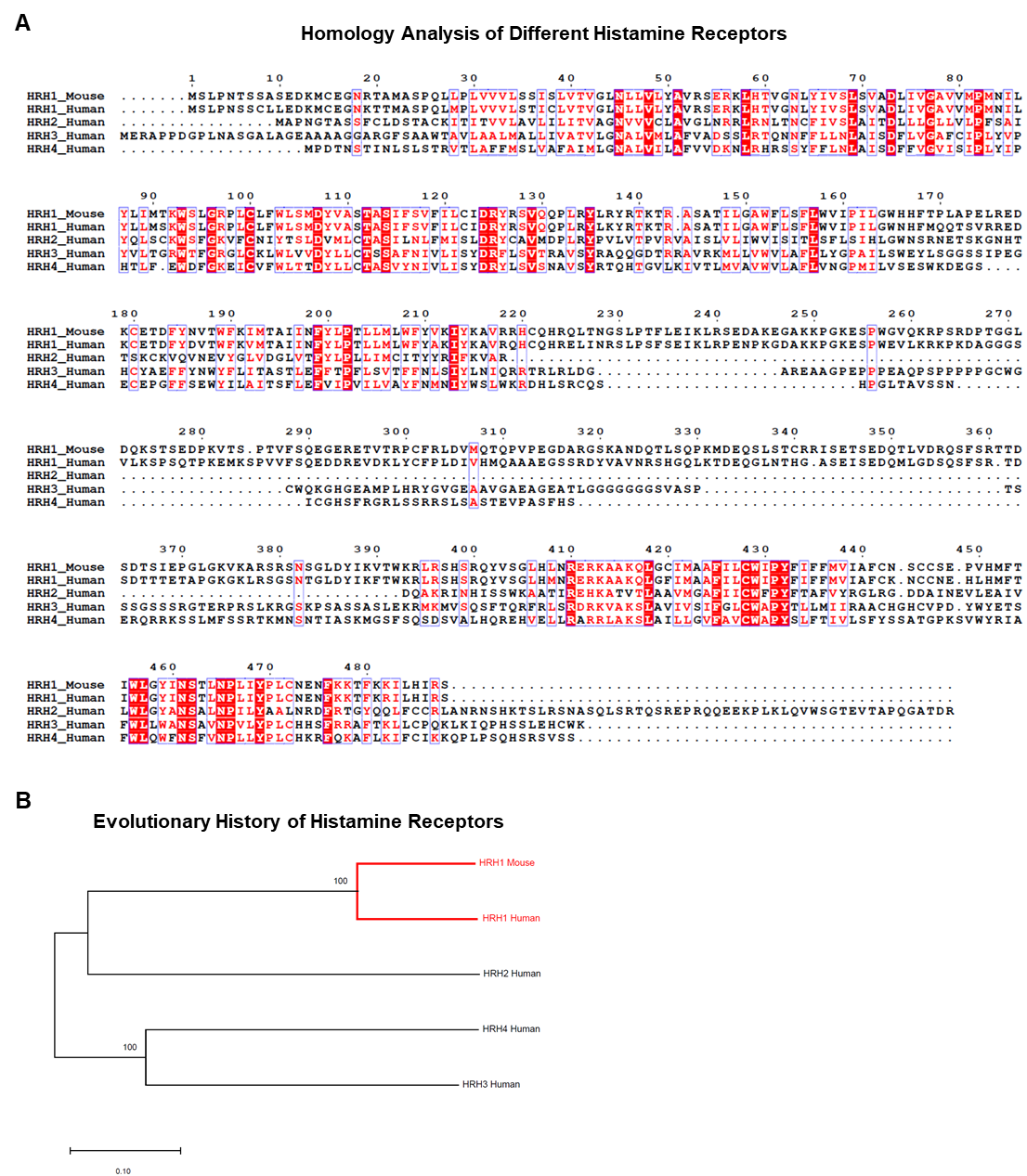


**Fig. S2. Histamine receptors are less conserved across different subtypes.**

(A) Homology analysis of different histamine receptors, including HRH1_Mouse, HRH1_Human, HRH2_Human, HRH3_Human and HRH4_Human. Sequences were aligned utilizing the ClustalW method in MEGA X software. Ambiguous positions were removed for each sequence pair. (B) The evolutionary history was inferred utilizing the neighbor-joining method based on the protein sequences of histamine receptors, including mouse HRH1, human HRH1, human HRH2, human HRH3 and human HRH4. The percentages of replicate trees in which the associated strains clustered together according to the bootstrap test (1000 replicates) are shown beside each branch. The tree was drawn to scale, with branch lengths in the same units as those of evolutionary distances used to infer the phylogenetic tree.

**Fig. S3**


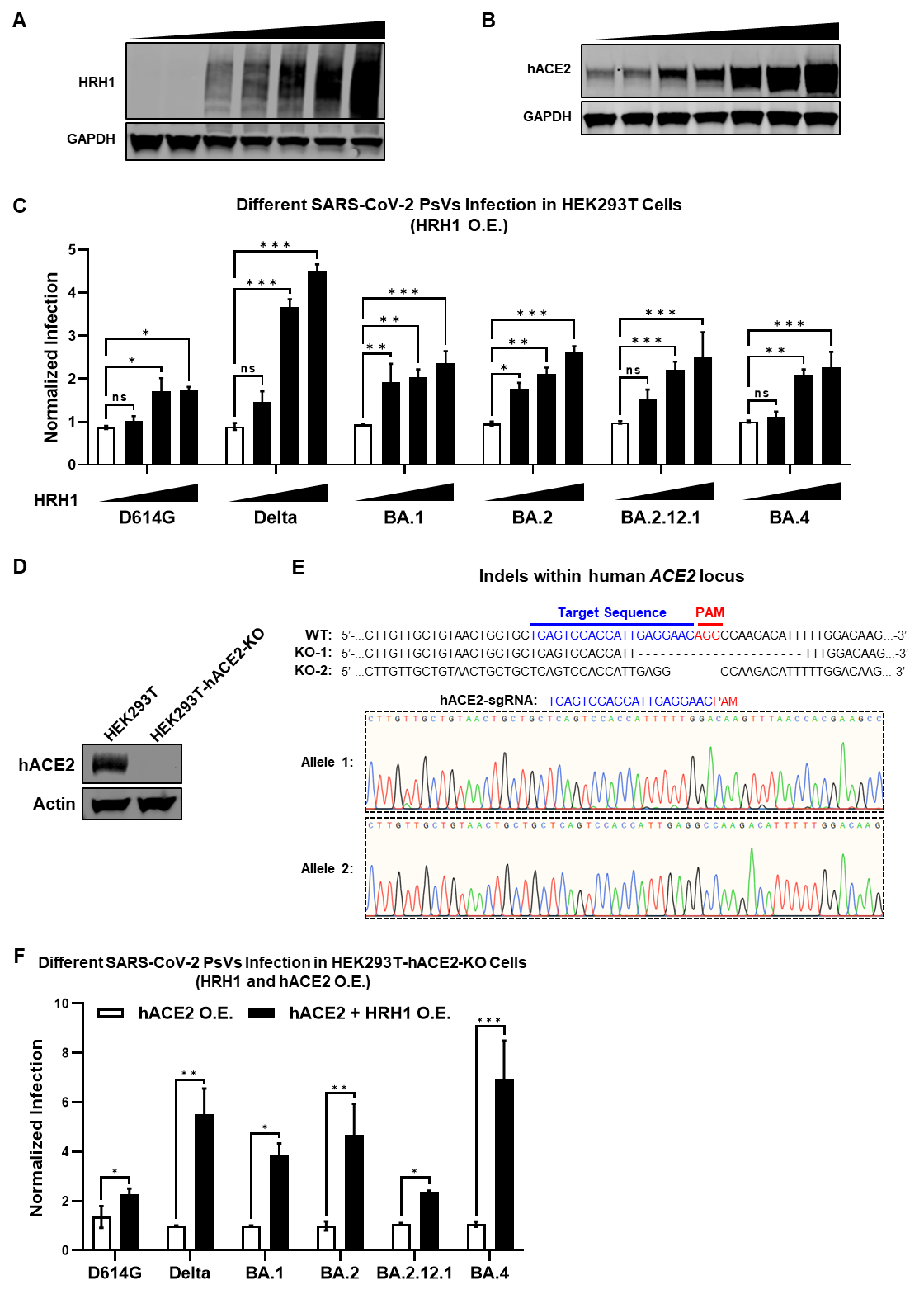


**Fig. S3. HRH1 enhanced ACE2-mediated SARS-CoV-2 entry.**

(A) HEK293T cells in 96-well plates were transfected with 0, 6.25, 12.5, 25, 50, 100 and 200 ng of HRH1-expressing plasmids, followed by infection with SARS-CoV-2 PsVs at 24 hpt. Cells were lysed, and western blot assays were performed with antibodies against HRH1 and GAPDH at 48 hpi. (B) HEK293T cells in 96-well plates were transfected with 0, 1.56, 3.125, 6.25, 12.5, 25 and 50 ng of hACE2-expressing plasmids, followed by SARS-CoV-2 PsV infection at 24 hpt. Another 48 h later, the cells were lysed and immunoblotted with antibodies against hACE2 and GAPDH. (C) HEK293T cells were transfected with 0, 1, 2 or 4 ng of HRH1-expressing plasmid. At 24 hpt, the cells were further infected with different SARS-CoV-2 PsVs, including D614G, Delta, BA.1, BA.2, BA2.12.1 and BA.4. After 48 hpi, the relative luciferase activities of the cells were measured. The infectivity of each combination was calculated by normalizing the luminescence units of each group to those of the 0 ng HRH1 group. (D) The efficiency of CRISPR-Cas9-mediated ACE2 knockout in HEK293T cells was evaluated by western blotting. (E) Sanger sequencing results of the HEK293T-hACE2-KO monoclonal cell line. Both alleles of the *ACE2* gene were mutated and harbored deletion indels. The DNA region in blue indicates the hACE2 sgRNA target sequence. (F) HEK293T-hACE2-KO cells were transfected with 2 ng of hACE2-expressing plasmids. Another group of cells was cotransfected with 2 ng of hACE2-expressing plasmids and 4 ng of HRH1-expressing plasmids. At 24 hpt, the cells were infected with SARS-CoV-2 D614G, Delta, BA.1, BA.2, BA.2.12.1 and BA.4 PsVs, respectively. Relative luciferase activities within each group were measured and calculated by normalizing the luminescence units of each group to those of the hACE2 expression-only group at 48 hpi. The data in (C) and (F) represent the mean ± SEM of biological triplicates. *P* values in (C) were calculated by two-way ANOVA with Dunnett’s multiple comparisons tests, while *P* values in (F) were calculated by Student's *t*-test. **P* < 0.05, ***P* < 0.01, ****P* < 0.001.

**Fig. S4**


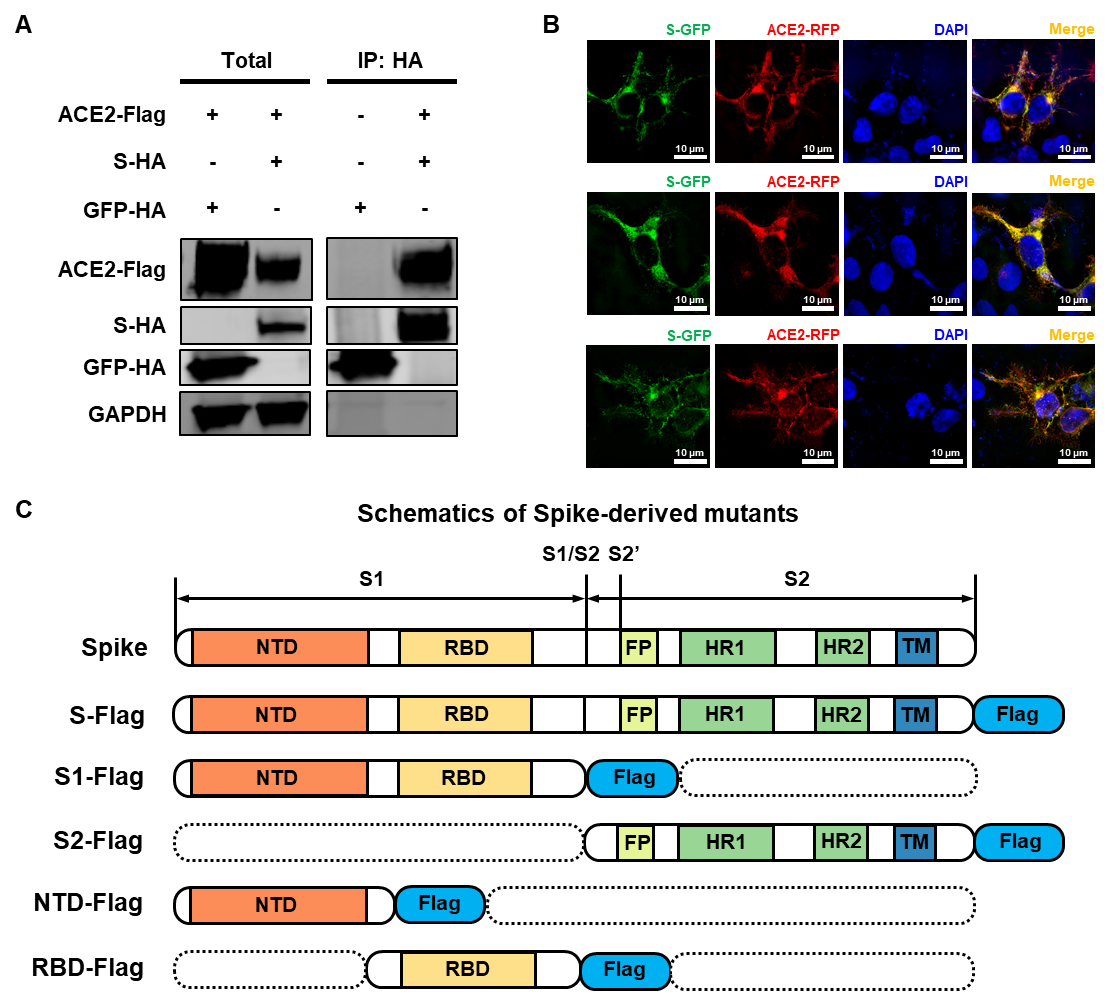


**Fig. S4. The localization of the spike protein and schematics of Spike mutants.**

(A) HA-tagged S and HA-tagged GFP were co-overexpressed with Flag-tagged ACE2 in HeLa cells. At 48 hpt, IP assays were conducted by lysing cells and enriching proteins with anti-HA beads. Both total and IP samples were subjected to western blot assays with antibodies against HA, Flag and GAPDH. (B) GFP-tagged S and RFP-tagged ACE2 were co-overexpressed in HEK293T cells, followed by SIM imaging at 24 hpt. DAPI was used to dye DNA. Three representative images were captured. (C) Schematics of spike-related mutants, including full-length S, S1, S2, the NTD and the RBD. Each mutant was conjugated with a Flag tag at its C-terminus. The scale bars in (A) represent 10 μm. At least three samples were harvested for SIM imaging.

**Fig. S5**


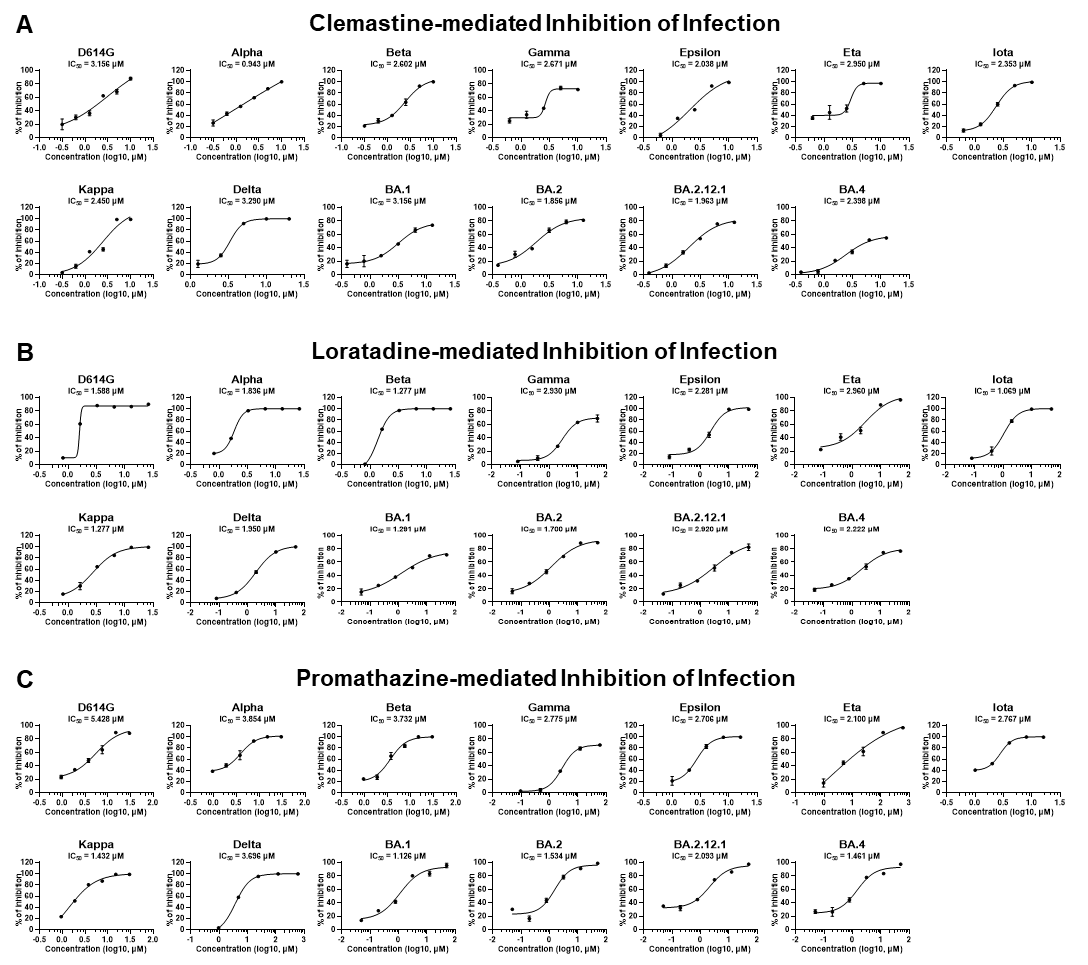


**Fig. S5. Antihistamine drugs inhibited SARS-CoV-2 mutant infection.**

(A) Clemastine at serially diluted concentrations was premixed with 13 different kinds of SARS-CoV-2 PsVs, followed by incubation with HEK293T-hACE2 cells. At 48 hpi, the cells were lysed, and luciferase activity was measured. The IC50 value for each viral mutant was calculated based on the relative luciferase activity at each drug concentration. (B-C) The IC50 values of loratadine and promethazine against 13 SARS-CoV-2 PsVs were measured and calculated as described in (A). The data in (A-C) are presented as the means ± SEMs of biological triplicates.

**Fig. S6**


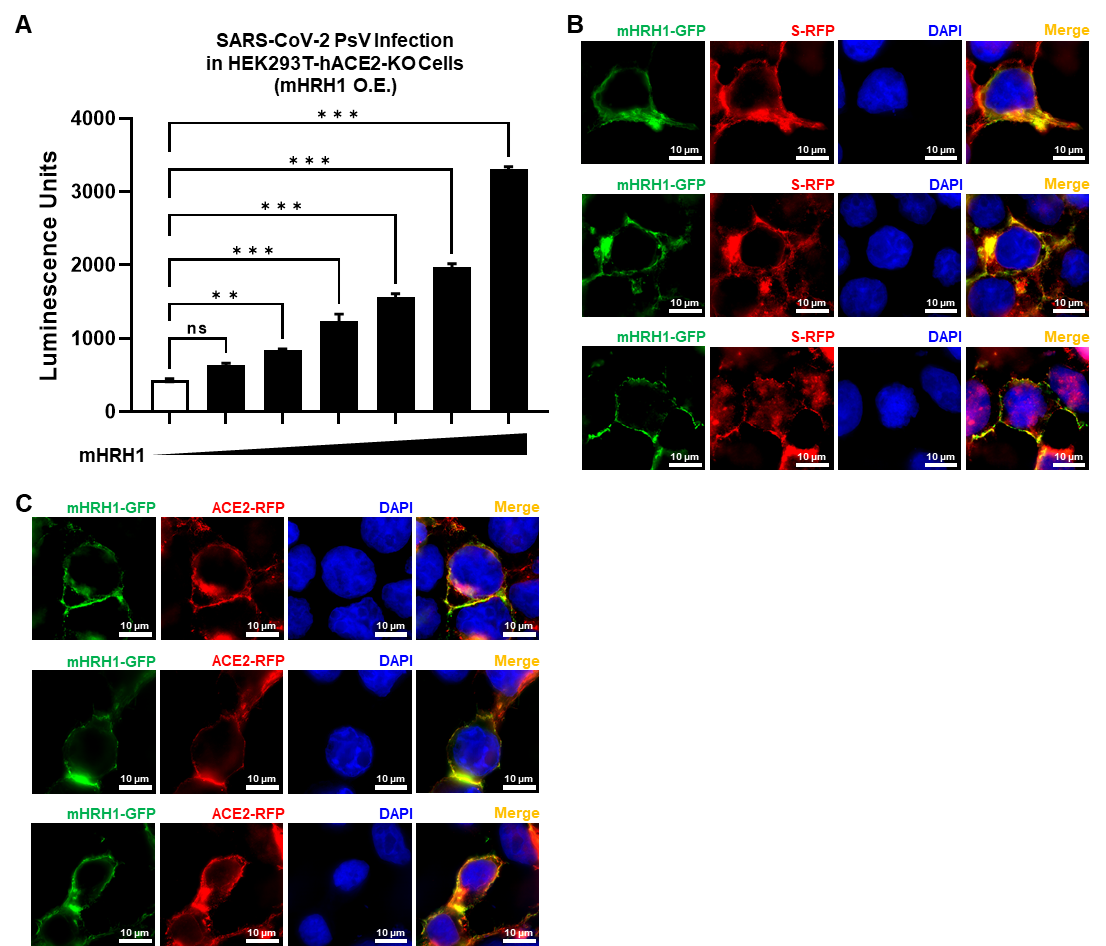


**Fig. S6. mHRH1 promoted SARS-CoV-2 entry and colocalized with the spike protein.**

(A) HEK293T-hACE2-KO cells were transfected with different amounts of mHRH1-expressing plasmids (0, 6.25, 12.5, 25, 50, 100 and 200 ng) and infected with SARS-CoV-2 D614 PsVs at 24 hpt. At 48 hpi, the cells were lysed, and luminescence was measured. (B) HEK293T cells were co-overexpressing with GFP-tagged mHRH1 and RFP-tagged SARS-CoV-2 S, followed by SIM imaging at 24 hpt. Genomic DNA was stained with DAPI. The data are shown as three representative colocalization images. (C) HEK293T cells were co-overexpressing with GFP-tagged mHRH1 and RFP-tagged ACE2. SIM imaging was conducted for transfected cells as described in (B). The data in (A) are presented as the means ± SEMs of biological triplicates. *P* values were calculated by one-way ANOVA with Dunnett's multiple comparisons test. **P* < 0.05, ***P* < 0.01, ****P* < 0.001. The scale bars in (B) and (C) represent 10 μm.
